# Changes to the Natural Killer Cell Repertoire after Therapeutic Hepatitis B DNA Vaccination

**DOI:** 10.1371/journal.pone.0008761

**Published:** 2010-01-18

**Authors:** Daniel Scott-Algara, Maryline Mancini-Bourgine, Hélène Fontaine, Stanislas Pol, Marie-Louise Michel

**Affiliations:** 1 Unité de Régulation des Infections Rétrovirales, Institut Pasteur, Paris, France; 2 Laboratoire de pathogenèse des virus de l'hépatite B, Institut Pasteur, Paris, France; 3 Institut National de la Santé et de la Recherche Médicale U845, Paris, France; 4 AP-HP, Service d'hépatologie, Hôpital Cochin, Université René Descartes Paris V, INSERM U 567, Paris, France; 5 AP-HP, Service d'hépatologie, Hôpital Cochin, Paris, France; Statens Serum Institute, Denmark

## Abstract

**Background:**

Improvements to the outcome of adaptive immune responses could be achieved by inducing specific natural killer (NK) cell subsets which can cooperate with dendritic cells to select efficient T cell responses. We previously reported the induction or reactivation of T cell responses in chronic hepatitis B patients vaccinated with a DNA encoding hepatitis B envelope proteins during a phase I clinical trial.

**Methodology/Principal Findings:**

In this study, we examined changes in the peripheral NK cell populations occurring during this vaccine trial using flow cytometry analysis. Despite a constant number of NK cells in the periphery, a significant increase in the CD56^bright^ population was observed after each vaccination and during the follow up. Among the 13 different NK cell markers studied by flow cytometry analysis, the expression of CD244 and NKG2D increased significantly in the CD56^bright^ NK population. The ex vivo CD107a expression by CD56^bright^ NK cells progressively increased in the vaccinated patients to reach levels that were significantly higher compared to chronically HBV-infected controls. Furthermore, modifications to the percentage of the CD56^bright^ NK cell population were correlated with HBV-specific T cell responses detected by the ELISPOT assay.

**Conclusions/Significance:**

These changes in the CD56^bright^ population may suggest a NK helper effect on T cell adaptive responses. Activation of the innate and adaptive arms of the immune system by DNA immunization may be of particular importance to the efficacy of therapeutic interventions in a context of chronic infections.

**Trial Registration:**

ClinicalTrials.gov NCT00988767

## Introduction

Natural Killer (NK) cells have recently been identified as crucial actors of innate host immunity in response to a variety of pathological challenges [1]. Their role in controlling pathogenesis induced by infection is dual and can occur through both cytokine/ chemokine secretion and antibody dependent or natural cytotoxic activity toward infected target cells. Human NK cells represent 5–20% of all circulating lymphocytes and based upon their cell surface density of CD56, two distinct populations of human NK have been identified [2]. Most of human NK cells have low-density expression of CD56 (CD56^dim^) and are the more cytotoxic subset. In contrast, CD56^bright^ subset that represents 10% of the NK cells has the capacity to produce abundant cytokines**.** NK cells also express several families of receptors including both inhibitory and activating receptors [1]. These receptors, by delivering inhibitory signals to NK cells, can prevent unwanted responses to normal cells that express a complete set of self-MHC molecules. Several studies reported distinct NK cell repertoire and/or NK cell ligand expression during viral infections and their correlation in either the control or the resistance against infections [3]. The cross talk between NK and antigen presenting cells influences efficiency of adaptive immune responses against virus, thus constituting a major link between innate and adaptive immune responses [3].

During the early phase of hepatitis B virus (HBV) infection, the activation of innate immunity (including NK cells able to produce large quantities of IFN-γ) seems to be an important factor determining the subsequent induction of adaptive immunity and ultimately the outcome of HBV infection [4], [5]. It now seems well established that the differences in adaptive immunity that characterizes chronically-infected patients and those with resolved infection are heavily influenced by immunological events during the initial phase of HBV replication. Activating innate immunity could thus be of major importance when attempting to control chronic infection. Recently, DNA-based vaccines have been proposed as a new tool to stimulate immune responses that are functionally exhausted during chronic viral infections [6]. In a previous report, we demonstrated that DNA vaccination could specifically activate T-cell responses in HBV-carriers with chronic active hepatitis not responding to current anti-viral therapies [7]. Plasmid DNA vaccines target antigen-presenting cells, including dendritic cells (DC), to induce T-cell responses [8]. They contain immunostimulatory CpG motifs which have been shown to stimulate the innate immune system via toll-like receptor (TLR) 9 [9]. CpG motifs augment NK cell activity indirectly by inducing the secretion of IL-12, IFN α/β and TNF-α [10], [11]. During DNA vaccination, the cross-talk between NK cells and DC could be essential to inducing an adaptive immune response. In the present study, we evaluated modifications to the NK cell repertoire during a therapeutic DNA vaccination trial conducted in chronically HBV-infected patients, and tried to correlate these modifications to the induction of an adaptive immune response.

## Methods

### Participants and Study Protocol

Chronic HBV carriers, all male, with a median age of 43 years, with biopsy-proven chronic hepatitis, active HBV replication and no decompensated liver disease, were enrolled in a phase I clinical trial focused on safety and whether DNA vaccination could restore T-cell responsiveness during chronic HBV infection. All patients were long-term HBV carriers, mostly contaminated during childhood, and had not responded to IFN-α and/or lamivudine therapy. The DNA vaccine was injected simultaneously into each deltoid muscle (1 mg total DNA) at months 0, 2, 4. Five out of nine patients received an additional injection at month 10. Blood samples were collected before and one month after each immunization. The protocol for this trial and supporting CONSORT checklist are available as supporting information; see Checklist S1 and Protocol S1.

The historical control group of HBV-infected patients consisted in 6 males and one female, with a median age of 45 years, with chronic hepatitis, active HBV replication (see Table 1). They were enrolled in a prospective study aiming at characterizing HBV-specific immune responses and all gave their informed written consent to participation (EUDRACT N° 2006-004172-12).

**Table 1:** Clinical parameters and NK phenotype of vaccinated (HB) or untreated (CAH) patients with chronic active hepatitis.

Patient^a^	HBeAg	anti-HBe	HBV DNA (pg/ml)	ALAT/ASAT (IU/ml)	% CD56^highc^
HB13	−	+	<2.5^b^	98/39	4
HB14	+	−	552	50/35	3
HB16	+	−	174	70/48	4
HB17	+	−	804	61/83	8
HB18	+	−	15428	227/166	2
HB21	+	−	10	222/87	4
HB22	+	−	1348	114/46	6
HB23	+	−	902	114/60	2
HB24	+	−	3968	46/28	4
CAH1	−	+	244	189/146	13
CAH2	−	+	88	198/111	1
CAH3	−	+	341	562/267	15
CAH4	+	−	3	61/41	9
CAH5	+	−	2200	73/65	5
CAH6	−	+	2	47/34	5
CAH7	−	+	3	60/44	5

a Data from HB patients were collected before DNA injections.

b HBV DNA from HB13 was repeatedly detected before inclusion.

c % of the CD56^high^ expression on NK cells as defined in Patients and Methods section.

### Ethics Statement

This study was approved by the local Ethics Committee and all study participants gave their informed, written consent to participation, in line with French ethical guidelines. Part of the results of the trial was previously reported [7]. Authorization was granted by the “Comité Consultatif de Protection des Personnes dans la Recherche Biomédicale de Paris Necker” on November 13, 2000, approved by the French authorities (AFSSAPS registration N° 010030) and Biomedical Research Directorate at Institut Pasteur. INSERM was the sponsor of the study.

### DNA Vaccine

The pCMV-S2.S DNA vaccine [12] was produced under GMP conditions by Qiagen GmbH (Hilden, Germany). This plasmid encodes the small (S) and middle (preS2+S) proteins of the HBV envelope (ayw subtype) under the control of the human cytomegalovirus promoter; it has been shown to induce hepatitis B surface (HBs)-specific T cells and anti-envelope antibodies in mice and chimpanzees [13], [14], [15]. DNA was formulated in endotoxin-free 0.9% NaCl to give a dose of 1 mg/ml.

### Phenotypic Studies of NK Cells

All phenotypic studies were performed on whole blood by four-color flow cytometry using an EPICS MCL II cytometer, and analyzed using System II software (Coulter, Margency, France). Lymphocytes were gated in FSC/SSC dot plot and CD3 positive lymphocytes were eliminated from the analysis by plotting lymphocytes in an FSC/CD3 expression dot plot. Negative CD3 cells were evaluated by the expression of CD56 and/or CD16. The proportion of total NK cells was defined as the % of CD3-CD56+CD16+ , CD3-CD56+CD16-, CD3-CD56-CD16+ cells in the lymphocyte population. NK cell receptor expression analysis was performed in CD3-CD56+CD16+/− lymphocyte population. The detection of natural cytotoxicity receptors (NCR) (NKp30, NKp44, NKp46), killer inhibitory receptor (KIR) (CD158a, 158b, 158e, 158i), CD161, NKG2A, NKG2C, NKG2D and CD244 was performed using monoclonal antibodies from Beckman Coulter and RD Systems (Paris, France). The study of NK cell receptors started at M1 because of limitations concerning the collection of blood samples. CD107a (BD Biosciences, Paris, France) staining was used as an indirect measure of cytotoxicity for cells that underwent degranulation [16].

A historical group of untreated HBV-infected patients with chronic active hepatitis (see Table 1) and healthy donors were studied as controls.

### IFN-γ Enzyme-Linked Immunospot (ELISpot) Assay

The IFN-γ ELISpot assay was used to analyze T cell responses to HBV peptides following the in vitro expansion of PBMC as previously described [17]. The response was considered positive if the median number of spot-forming cells in triplicate wells was at least twice that in control wells containing irrelevant peptide, and at least 5 spots were detected per 2×10^5^ PBMC after background subtraction.

### Statistical Analysis

Analyses were performed using Prism software (GraphPad Software Inc., San Diego, CA) using the nonparametric Kruskall-Wallis test followed by Dunn's post-test and a Mann-Whitney test to compare two unpaired groups. A *p* value <0.05 was deemed statistically significant. Correlations between viral load, ALT and the % of CD56^bright^ NK cells were analyzed using the Pearson's correlation coefficient (r).

## Results

### DNA Vaccination Induces Modifications in NK Cell Subsets in Whole Blood from HBV Carriers

**Figure 1:**
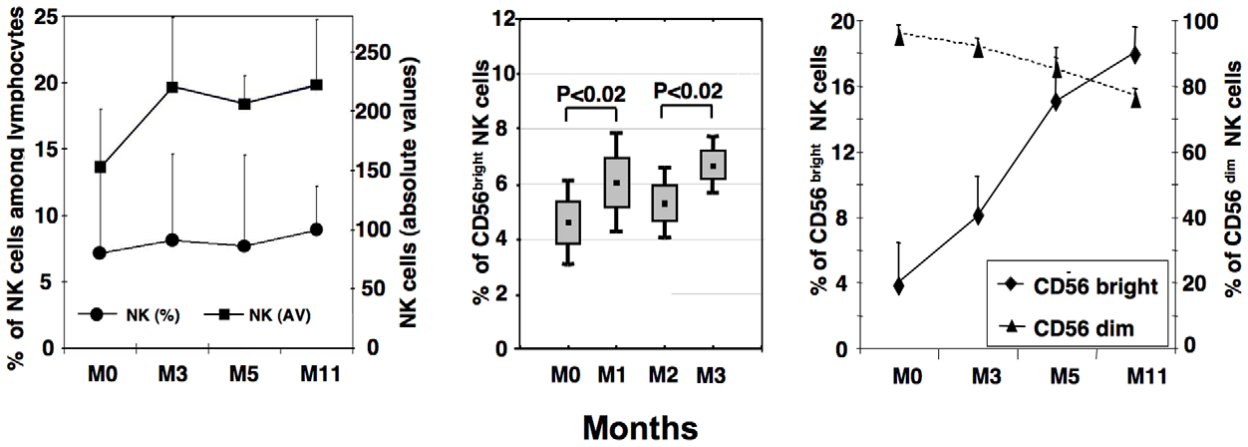
Follow-up of NK cells and NK subsets in whole blood during HBV DNA vaccination. Absolute values (AV) of NK cells/microL of blood and percentages of NK cells amongst lymphocytes (left panel). Increases in the percentages of CD56^bright^ after the first and second vaccine injections are shown for 5 patients (middle panel). Follow-up of CD56^bright^ and CD56^dim^ populations among NK cells (right panel). Blood samples were collected before the first vaccine injection (M0) and one month after the first (M1), the second (M3), the third (M5) and the fourth (M11) DNA vaccine injections. Analysis performed at M11 included blood samples from the 9 patients whether or not they received the fourth DNA injection. Results are shown as mean ± SE and percentile 95.

DNA vaccines have been shown to activate an adaptive immune response in rodents and humans [10]. However, the activation of innate immunity by DNA vaccines has never been tested in humans. We therefore studied specific changes to peripheral NK cell populations during the follow-up of nine HBV-infected individuals vaccinated with DNA encoding HBV envelope proteins. As shown in **Fig. 1**, the mean percentage of NK cells amongst lymphocytes and the absolute number of NK cells remained stable over time (**Fig. 1A, left panel**). However, within NK cells, a significant increase in the proportion of CD56^bright^ NK cells defined as CD56^bright^ CD16- was observed one month after the first (M1 vs. M0, p<0.02) and second (M3 vs. M2, p<0.02) vaccine injections, suggesting that this population was triggered by DNA vaccination (**Fig. 1A** **, middle panel**). The low number of patients (i.e. 5) receiving the fourth DNA injection at month 10 did not make possible to show a significant increase in the proportion of CD56^bright^ NK cells between M10 and M11. Nevertheless, the percentage of the CD56^bright^ population significantly increased during the entire course of the vaccination protocol (p<0.005 M11 vs. M0) (**Fig. 1A, right panel**). The frequency of the CD56^dim^ population defined as CD56^dim^CD16+/− among NK cells accordingly decreased. No change in the % of CD3- CD56- CD16+ NK cells was found in the studied patients compared to healthy controls (not shown).

### Modifications in Peripheral Blood NK Cell Repertoire from HBV Carriers after DNA Vaccine Injection

**Figure 2:**
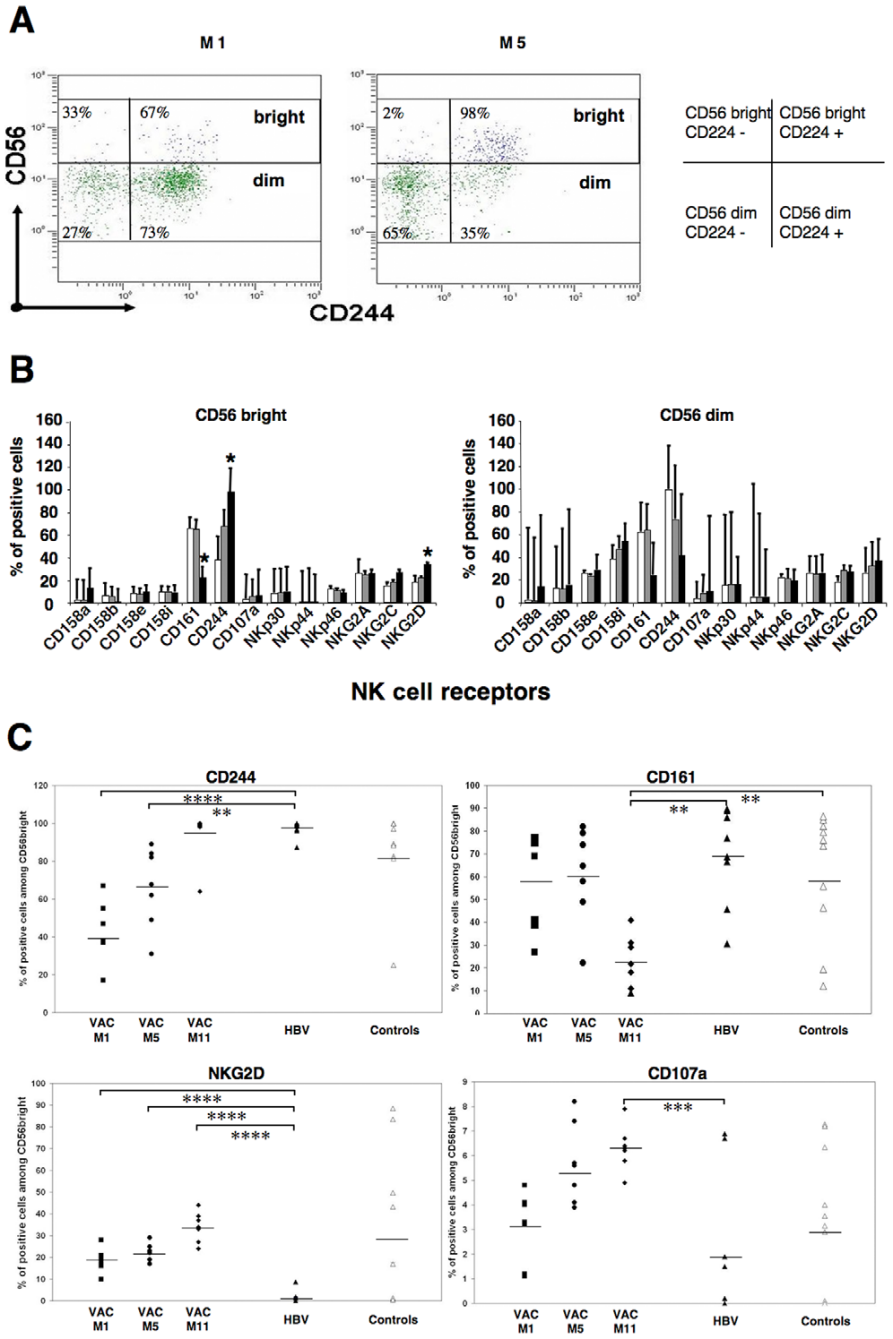
Flow cytometry analysis of NK cell repertoire. **A**. A typical analysis is shown of NK cell subsets and receptor expression in blood collected at two time points (M1 and M5) from one patient. Dot plots are gated in NK cells as defined in Patients and Methods section. Subsets of NK cells are defined by the level of CD56 marker expression, as indicated (dim, bright). CD244 receptor expression in the two subsets is given as example. Percentages of cells expressing or not the CD244 receptor in CD56**^bright^** and CD56**^dim^** subsets are indicated. **B.** Follow-up of NK cell marker expression in the CD56^bright^ (left panel) and CD56^dim^ (right panel) subsets. Percentages of the different markers are shown for each subset. Results are shown as mean ± SE from seven patients. Blood samples collected at M1 (empty columns), M5 (grey columns) and M11 (black columns) time points were analyzed. Significant differences are indicated by * p<0.05. **C.** Follow-up of NK cell markers and CD107a expression in the CD56^bright^ subset. Percentage of CD56^bright^ NK cells expressing CD244 (upper left panel), CD161 (upper right panel), NKG2D (lower left panel) or CD107a (lower right panel) marker is shown for individual patients. Mean values are represented by bars. Blood samples from 7 vaccinated patients collected at M1 (Vac-M1), M5 (Vac-M5) and M11 (Vac-M11) time points were analyzed. Blood samples from 7 non-vaccinated HBV-infected patients with chronic active hepatitis B (HBV) and from 9 healthy donors (controls) were analyzed as controls . P values are indicated on the graph by stars: **: *p* = 0.003; *** *p* = 0.001; **** *p* = 0.0001.

We next tested whether modifications to subsets of NK cells were accompanied by changes to the repertoire of NK cells. We analyzed the expression of NK cell markers including NCR and KIR in the two major sub-populations of NK cells; a typical example is shown in **Fig. 2A** **.** Comparisons of blood samples taken at months 1 and 5 showed a remarkable increase in CD244 receptor expression in the CD56^bright^ subset.

Cell analysis of 13 different NK cell markers from 7 vaccinated patients was performed at three different time points (M1, M5 and M11; **Fig. 2B**)**.** As controls, NK cell markers analysis was performed on cells from a group of HBV-infected patients with comparable clinical and virological profiles (see **Table 1**) and on cells from healthy individuals. Although variations in the expression of different receptors did exist in the studied patients, the expression of three NK cell markers (CD244, NKG2D and CD161) was significantly modulated at M11 in the CD56^bright^ NK cell subset from vaccinated patients (**Fig. 2B** left panel). A significant increase in CD244 and NKG2D expression was observed in the CD56^bright^ NK cell subset at the later time point (*p<0.05*; **Fig. 2B left panel**). For CD244 expression, a significant difference was observed for CD56^bright^ NK cells from vaccinated patients analyzed at M1 and M5, compared to NK cells from chronically HBV-infected control patients (*p = 0.0001* and *p = 0.003*, **Fig. 2C** **upper left panel**). NKG2D expression was significantly increased on CD56^bright^ NK cells from vaccinated patients at M1, M5 and M11 compared to NK cells from chronically HBV-infected control patients (*p = 0.0001,* **Fig. 2C** **lower left panel**). A significant decrease in CD161 expression was observed in the CD56^bright^ NK cell subset (**Fig. 2B left panel** **)** at M11 for vaccinated patients. At this later time point CD161 expression on CD56^bright^ NK cells from vaccinated patients was significantly lower than on cells analyzed from chronically HBV-infected patients or from healthy controls (*p = 0.003,* **Fig. 2C** **upper right panel**).

We also assessed the ex vivo expression of the degranulation marker CD107a, which represents an indirect measurement of the cytotoxic capacity for T cells or NK cells. Expression of CD107a on CD56^bright^ NK cells from vaccinated patients at M5 and M11 was significantly higher compared to chronically HBV-infected patients (*p = 0.07* and *p = 0.001* respectively; **Fig. 2C** **lower right panel**).

In the CD56^dim^ population, non-significant changes were observed in NK cell receptor expression **(** **Fig. 2B, right panel** **).** These results suggest that the CD56^bright^ NK population is specifically targeted by DNA vaccination.

### Activation of the CD56^bright^ NK Cell Subset Does Not Correlate with HBV Viral Load

An early rise in circulating NK cells was found in acutely HBV-infected individuals concomitant with the peak of HBV replication with a subsequent decrease at the time of HBV-DNA drop [18]. We asked whether the increase in the CD56^bright^ NK population could be modulated by changes in viral load or flares in hepatitis. We compared data from 9 patients before DNA vaccination (M0) and 7 patients with similar virological and clinical profiles (**Table 1**). However, no correlation was found (r = −0.0559) between HBV viral load and the % of CD56^bright^ NK cells. No correlation (r = −0.0635) between ALT and the % of CD56^bright^ NK cells was found either. Thus the observed increase in the CD56^bright^ NK cell population could not be linked to viral load or flares that are frequently observed in patients with chronic active hepatitis.

### IFN-γ-Secreting T Cell Responses Correlate with Activation of the CD56^bright^ NK Cell Subset

**Figure 3:**
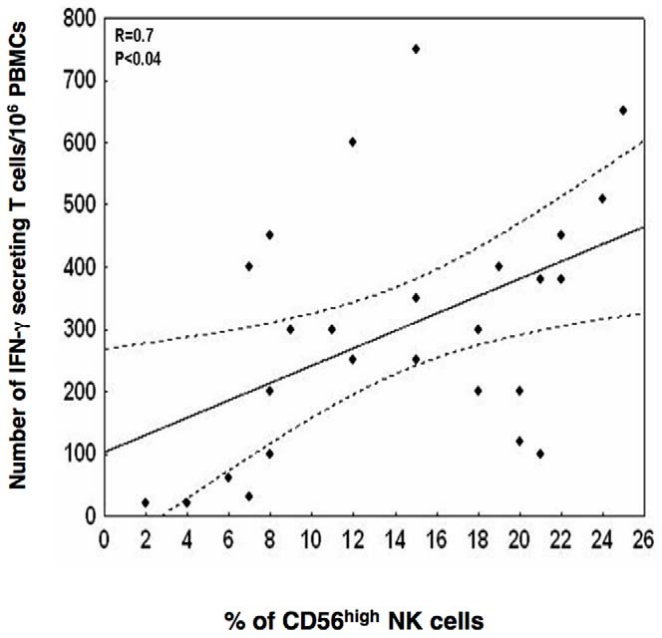
Correlation between IFN-γ-secreting HBV-specific T-cell responses and % of CD56^bright^ NK cells. HBV S-specific T-cell responses in patients detected using IFN-γ ELISpot assay are expressed as the number of spot-forming cells/million PBMC and plotted against percentages of CD56^bright^ NK cell subsets quantified at the same time points during the follow-up of HBV DNA vaccination.

We previously reported that administration of a DNA vaccine encoding two HBV envelope proteins to chronic HBV-carriers could induce or reactivate IFN-γ-producing T cell responses specific to HBV preS2 and S antigens [7]. In this study, S-specific responses were found in all patients, although this response was already detectable for two of them before DNA-injection. PreS2-specific responses were detected in 5 out of 9 patients [7]. We then tried to determine whether the NK cell repertoire modifications observed in the context of HVB-DNA vaccination might be linked to induction of the adaptive immune response. For this purpose, we used the nonparametric Kruskall-Wallis test followed by Dunn’s post-test to compare the number of IFN-γ-secreting T cells detected by the ELISPOT assay and the corresponding percentage of CD56^bright^ NK cells within the NK cell population. These analyses were performed on S-specific T cell responses as only these responses were found significantly higher in patients receiving DNA injections than in unvaccinated patients (p = 0.028, [7]). As shown in **Fig. 3** **,** the proportion of CD56^bright^ NK subset was positively correlated with detectable T cell responses against HBV-S peptides, tested using the IFN-γ ELISPOT assay (r* = *0.7, p<0.04). In addition, a stronger positive correlation was observed when combined preS2- and S-specific IFN-γ-secreting T cell responses were analyzed (r* = *0.92, p<0.01). This result suggests that modifications to the NK cell repertoire might have modulated the adaptive immune response in the context of DNA vaccination.

## Discussion

Priming of protective T cell responses has been shown in different models of virus infection to be deeply affected by the cross-talk between NK and DCs, where NK cells play a key regulatory function essential for DC maturation and subsequent T cell priming [19]. The activation of NK cells during therapeutic intervention may therefore be important to the clearance of infection. Here, we have demonstrated that the DNA-based vaccination of HBV chronic carriers not only activated detectable HBV-specific T cells in the periphery but also induced an increase in a particular subset of NK cells. The NK cell increase was correlated with the number of HBV-specific IFN-γ-secreting T cells activated by the DNA vaccine.

During our study, we used a DNA vaccine encoding HBV envelope proteins (HBsAg) to activate or reactivate HBs-specific T cells which were non-responsive during chronic HBV infection. The activation of immune cells could be linked either to the antigen encoded by our DNA vaccine or to the DNA vaccine backbone. CpG sequences contained in bacterial DNA may trigger the maturation of DC and other immune cells expressing TLR9, that in turn secrete cytokines. In humans, TLR9 seems to be expressed exclusively by pDCs and B cells, and its stimulation induces the production of type I IFN and IL-12 [9]. These cytokines have been shown to activate in vivo antiviral NK cell function in mice [20]. Reports of TLR9 expression by NK cells are conflicting but suggest that some NK subsets may express sufficient TLR 9 for them to be directly activated by its ligand [21]. In addition, the dual activation of DC and NK cells might facilitate TLR-mediated activation of NK cells through DC-secreted cytokines [22].

Although direct evidence is lacking with respect to the role of NK cells during natural HBV infection, experiments in chimpanzees have suggested that the initial host response to HBV is primarily sustained by NK and NK T cells [23], [24]. In addition, the analysis of NK cell frequencies in patients studied during the incubation phase of acute hepatitis B has shown an early rise in circulating NK cell levels concomitant with HBV replication and preceding the peak of HBV-specific CD8 T cells [18]. Recently, detailed studies on early kinetics of innate and adaptive immune responses during initial phases of HBV infection have been reported. These studies show an inhibition of NK cell activation with a reduction in anti-viral cytokine secretion at the time of peak HBV viremia [25]. The development of NK and NKT cell response was correlated to initial viral containment and to the subsequent induction of adaptive immune responses [5]. For patients with chronic HBV infection, Oliviero et al. recently reported a profound alteration in NK cell functions, with a defective NK cytokine production in the context of a relatively normal NK cytolytic activity [26]. Despite the lack of samples at baseline for NK cell repertoire, our results suggest that the CD56^bright^ NK population and its repertoire is specifically targeted by DNA vaccination in chronic HBV-carriers. This observation is particularly relevant regarding the modulation of CD244 receptors which may act as inhibitory or activating receptors, depending on the density of its expression on NK cells. CD244 has been shown to be internalized after stimulation and/or ligand interaction [27]. Compared to control patients CD244 expression was initially found down-regulated in vaccinated patients, whereas at the last time point of the follow up CD244 expression returned to basal level. CD244 was previously found to be up-regulated in vivo by an injection of CpG [28]. This up-regulation may contribute to increasing NK survival and/or CD8 specific responses. One can speculate that the later increase may be due to continuous stimulation of NK cells and to homeostasis mechanisms as shown in other models [29]. Modifications to NK cell receptors could be important to the outcome of HBV infection, either by tuning the T cell response or promoting pathways for liver damage [30].

In other models such as hepatitis C, the role of NKG2A and its ligands has been shown to inhibit cytokine production and the cytolytic activity of antigen-specific T cells [31], [32]. In our study, no modifications were observed to the expression of NKG2A on NK cells. However, expression of the NKG2D receptor gradually increased after the first DNA injection in our vaccinated patients. Its expression was significantly higher at the end of the vaccine protocol compared to non-vaccinated HBV-infected controls. NKG2D functions both as an activating and a co-stimulatory receptor having a fundamental role in the surveillance of microbial infections and cancer [33], [34]. In a mouse model, an increased expression of NKG2D after DNA vaccination was correlated with an improved NK and CD8 T cell response to tumor cells, irrespective of the expression of NKG2D ligands [35], [36]. In another study, the increased expression of NKG2D was correlated with more pronounced immune disease affecting the liver in an HBsAg transgenic mouse model [37]. Moreover, ex vivo CD107a expression by CD56^bright^ NK cells progressively increased in our vaccinated patients to reach levels that were significantly higher compared to chronically HBV-infected controls. Increase in both NKG2D and CD107a expression on NK cells during the vaccination protocol are suggestive of an enhanced cytolytic capacity. Although a 10−15 fold increase in transaminase activity was observed in two of our patients, we did not observe any major liver complications during their follow-up [7].

CD161 engagement appears to have a complex role in the modulation of immune functions with both activating and inhibitory effects [38]. CD161 was involved in triggering of NK cell cytotoxicity towards a tumor cell line [39]. In our study, the percentage of CD56^bright^ NK cells expressing CD161 decreased at the latter time point. Knowing that down regulation of CD161 expression is related to the secretion of IL-2, one can speculate that IL-2 secretion by the HBV-specific T cells may have contributed to the observed decrease in CD161 expression [39].

We also found that the activation of IFN-γ-secreting HBs-specific T cells correlated with the increase in NK cells expressing high levels of CD56. Several hypothesis can be put forward to explain such observation. Recent reports suggest an important role for NK cells in CD8+ T cell responses. On one hand, recognition and killing of target cells by NK cells represents an important pathway for the generation of robust antigen-specific CD8+ T and humoral responses, in providing apoptotic cells as a source of antigen for cross presentation to DC during viral infection [40]. On the other hand, NK-cell activation induces release of IFN-γ, which in turn can cause maturation of DC and polarize T-cell responses [41]. Interestingly, in addition to the induction of HBV envelope-specific T cells by the vaccine, an increase in T cells specific to a promiscuous capsid-derived HBV epitope was found in some patients during follow-up, although this increase was not significant [7]. Because the vaccine used during this study encoded envelope but not capsid proteins, this increase had previously been attributed to a bystander effect of cytokines secreted by specifically activated T cells. Based on our current findings, the amplification of capsid-specific T cells might result from the increase observed in the CD56^bright^ subset of NK cells. IFN-γ is considered to be the prototypic NK-cell cytokine and its production by NK cells is known to shape the Th1 immune response, activate APCs to further up-regulate MHC class I expression [41] and exert an anti-viral effect on HBV-infected hepatocytes [4]. It was recently shown that both CD56^dim^ and CD56^bright^ NK cell subsets from patients with chronic HBV infection display significantly lower INF-γ and TNF-α production [26]. We do not know whether these functions were restored in our vaccinated patients because this was not assessed in the present study due to the limited size of available samples.

Altogether these observations are in favor of the activation of both innate and adaptive immune responses during DNA vaccine immunotherapy in chronic HBV carriers. This could have been mediated directly via NK-T cell interactions and/or NK-DC crosstalk. Activation of the innate and adaptive arms of the immune system, and crosstalk between lymphocytes, may be of particular importance to the efficacy of therapeutic interventions in a context of chronic infections. Further studies would include triggering both arms of the immune response. Decreasing viral load was proposed to achieve a better restoration of T cell functions during vaccine therapy. However this is currently a matter of debate as restoration is only transient [42]. Improving DNA vaccines by inserting a sequence involving NKG2D receptor-ligand interaction was shown to enhance innate and adaptive immune responses [36]. Targeting NK cells by combining DNA vaccine and immune modulators such as adjuvants (TLR agonists) and/or cytokines (e.g. IL-2, IL-15 or IL-21) could also be an exciting option as recently proposed in cancer immunotherapy [43].

## Supporting Information

Checklist S1 CONSORT Checklist
(0.19 MB DOC)

Protocol S1 Trial Protocol
(2.16 MB PDF)
