# Supplementary material for: Changes to the Natural Killer Cell Repertoire after Therapeutic Hepatitis B DNA Vaccination
Source: PLoS One. 2010 Jan 18;5(1):e8761. doi: 10.1371/journal.pone.0008761 (PMC2807462; doi:10.1371/journal.pone.0008761)
Supplement: Protocol S1 — Trial Protocol (2.16 MB PDF) [file pone.0008761.s002.pdf]

PROJET DE RECHERCHE BIOMÉDICALE

Vaccinothérapie spécifique par ADN nu au cours des hépatites chroniques B

Essai clinique utilisant un OGM

Promoteur : **INSERM**

Investigateur coordonnateur :

Dr Hélène Fontaine (Unité Inserm 370)
Service d'Hépatologie
Hôpital Necker-Enfants Malades - Paris

Lieux de réalisation de la recherche :

Service d'Hépatologie
Hôpital Necker-Enfants Malades - Paris

Service Hépato-Gastroentérologie
Hôpital Saint-Joseph - Marseille

Service Hépato-Gastroentérologie
Hotel Dieu - Lyon

Version Décembre 2000

PROJET DE RECHERCHE BIOMÉDICALE

Vaccinothérapie spécifique par ADN nu au cours des hépatites chroniques B

Essai clinique utilisant un OGM

<u>Promoteur</u> : INSERM

<u>Investigateur coordonnateur</u> :

Pr Christian Bréchot (Unité INSERM 370)
Service d'Hépatologie
Hôpital Necker-Enfants Malades - Paris

<u>Lieu de réalisation de la recherche</u> :

Service d'Hépatologie
Hôpital Necker-Enfants Malades - Paris

Décembre 2000

SOMMAIRE

GENERALITES :

1.1 Renseignements administratifs p 2

1.2 Produits utilisés dans l'essai p 3

1.3 Description du protocole p 4

1.4 Recueil de l'information, suivi et audit p 7

1.5 Consentement éclairé p 7

1.6 Lieu de production et de contrôle, lieux d'administration et de contrôle p 8

I RENSEIGNEMENTS RELATIFS A L'OGM : CONFINEMENT ET EVALUATION DES RISQUES DE DISSEMINATION.

I.A Renseignements relatifs à l'OGM p 9

I.B Renseignements relatifs aux interactions entre les OGM, l'environnement et l'entourage p 11

II RENSEIGNEMENTS PHARMACEUTIQUES ET BIOLOGIQUES

II.1 Brève présentation de la stratégie thérapeutique proposée p 12

II.2 Construction de l'OGM p 12

II.3 Production et contrôles de routine p 15

II.4 Utilisation du produit p 16

II.5 Stockage p 17

III ELEMENTS DE PHARMACO-TOXICOLOGIE

III.1 Données de pharmaco-toxicologie sur le gène d'intérêt p 18

III.2 Données de pharmaco-toxicologie sur les autres gènes p 18

III.3 Données de pharmaco-toxicologie sur l'OGM p 18

III.4 Données de pharmaco-toxicologie du produit administré p 19

IV ESSAI CLINIQUE

IV.1 Rationnel p 24

IV.2 Objectifs p 39

IV.3 Patients p 39

IV.4 Traitements p 41

IV.5 Critères d'évaluation p 42

IV.6 Déroulement de l'essai p 44

IV.7 Suivi à court et à long terme de l'essai p 45

CALENDRIER DE SUIVI p 47

REFERENCES p 48

ANNEXE O : Notice d'information et formulaire de consentement p 53

GENERALITES

1.1. Renseignements administratifs

1.1.1. Identité du promoteur : **INSERM**

1.1.2. Identité des investigateurs :

Investigateur coordonnateur :

Pr Christian Bréchot
Service d'Hépatologie
Hôpital Necker-Enfants Malades
149, rue de Sèvres cedex
75743 Paris cedex 15
Tel : 01 44 49 51 26 - Fax : 01 44 49 51 65

INSERM U. 370
Faculté de Médecine Necker
156, rue de Vaugirard
75730 Paris cedex 15
Tel : 01 40 61 56 44 - Fax : 01 40 61 55 81

Investigateurs :

Pr Stanislas Pol
Service d'Hépatologie
Hôpital Necker-Enfants Malades

Dr Hélène Fontaine
Service d'Hépatologie
Hôpital Necker-Enfants Malades

1.1.3. Identité des chercheurs associés :

Dr Marie-Louise Michel
INSERM U. 163
Institut Pasteur
25-28, rue du Dr Roux
75724 Paris cedex 15
Tel : 01 45 68 88 49 - Fax : 01 45 68 89 43

Pr Pierre Tiollais
INSERM U. 163
Institut Pasteur
25-28, rue du Dr Roux
75724 Paris cedex 15
Tel : 01 45 68 88 20 - Fax : 01 45 68 89 43

1.1.4 CCPPRB saisi du projet

CCPPRB Necker-Enfants Malades.

1.2. Produits utilisés dans l'essai

1.2.1 Dénomination du produit

Plasmide bactérien recombinant pCMVHB-S2.S (également référencé dans les publications scientifiques et par la suite comme PCMV-S2.S) codant pour deux des protéines d'enveloppe du virus de l'hépatite B (VHB). La petite protéine est codée par le gène S et la protéine moyenne est codée par la région préS2 et par le gène S du VHB. Ces deux régions codantes sont sous le contrôle du promoteur des gènes précoces du cytomégalovirus.

Ce plasmide a été déposé sous le nom PCMVHB-S2.S à la Collection Nationale de Cultures de Micro-organismes (CNCM) sous le numéro I-1410 le 22/04/1994.

1.2.2 Brève présentation de la stratégie thérapeutique proposée

1.2.2.a Gène d'intérêt

Le vaccin utilisé sera constitué d'un plasmide bactérien recombinant produit dans les bactéries *E.coli* DH5 alpha. Cet ADN sera purifié par chromatographie sur colonne échangeuse d'ions (technique Qiagen). Au cours de cette procédure de purification l'ADN n'entrera en contact avec aucun agent toxique tel que le phénol, le chloroforme ou le bromure d'éthidium. La production sera réalisée conformément aux normes cGMP en accord avec les recommandations US 21 CFR. La technologie de purification proposée a été approuvée par les autorités concernées pour des essais cliniques en Europe (Medicine Control Agency MCA en Angleterre) et aux USA (FDA, drug master file type II: BB-MF 6224 et BB-MF 7882, voir annexe I.2).

La pureté de l'ADN sera contrôlée par HPLC, par recherche d'ADN génomique de E.coli, d'ADN simple brin et linéaire, d'ARN, d'endotoxines et de protéines bactériennes.

Une recherche de viabilité bactérienne sera effectuée (Bioburden assay). Voir annexe I.1 (exhibit II contrôle de qualité).

Le plasmide recombinant utilisé pCMV-S2.S comprend une partie permettant sa multiplication dans *E.coli* (origine de réplication et gène de résistance à l'ampicilline) et une partie permettant l'expression des gènes d'enveloppe du VHB.
- La partie bactérienne du vecteur (fragment BamH1- BssHII) est dérivée du plasmide pcDNA 3 (Invitrogen).
- Les deux protéines d'enveloppe VHB sont exprimées à partir d'un fragment du génome viral contenant le domaine préS2 et le gène S. Les signaux de polyadénylation des ARN messagers sont fournis par une séquence non traduite du génome VHB contenant également un signal d'export nucléaire des mARN. Le fragment d'ADN VHB utilisé est compris entre les sites SauI et Bgl II du VHB de sous type ayw. Le promoteur utilisé pour l'expression des gènes codant pour les protéines d'enveloppe est celui des gènes précoces du cytomégalovirus. Ce plasmide a été décrit dans un article scientifique *(1)* ; il a été utilisé avec succès pour induire des anticorps et une réponse cellulaire cytotoxique chez la souris, le lapin, le Macaque Rhésus et le chimpanzé (voir III.4.1.1.4).
Le plasmide pCMV-S2.S sera utilisé à visée thérapeutique.

1.2.2.b Système de transfert

Vaccination par ADN nu injecté par voie intramusculaire in vivo.

1.2.2.c Modalités d'administration

Injection intramusculaire sans adjuvant ni facilitateur de l'ADN en solution dans du chlorure de sodium à 9 pour mille (1mg/ml) : quatre injections d'un milligramme d'ADN réparti pour moitié entre les deltoïdes droit et gauche.
Intervalle de deux mois entre les trois premières injections qui seront suivies au dixième mois d'une injection de rappel. Ce protocole d'injection de doses fortes et répétées nous parait nécessaire pour l'obtention d'une réponse immunitaire stable *(2)*.

1.3 Description du protocole

1.3.1 Type d'étude

1.3.1 a : Il s'agit d'un essai de phase I. Dans un second temps, en fonction de la tolérance, un essai de phase II sera envisagé.

1.3.1 b : Il s'agit d'une recherche avec bénéfice individuel direct dans la mesure où, bien qu'il s'agisse d'une étude de phase I, les patients qui seront vaccinés pourraient bénéficier directement de l'effet de ce vaccin sur la multiplication du virus de l'hépatite B.

1.3.1 c : Il s'agit d'une étude monocentrique qui, en fonction des résultats de la tolérance, deviendrait multicentrique.

1.3.1 d : L'étude est non comparative.

1.3.1 e : L'étude est non randomisée.

1.3.1 f : Il n'y a pas d'évaluation en "aveugle".

1.3.1 g : Le recrutement prévu est de 10 patients.

<u>1.3.2. Justification de l'étude</u>

1.3.2 a : Il s'agit d'une étude à visée thérapeutique.

1.3.2 b : L'indication thérapeutique est l'amélioration du traitement des infections par le virus de l'hépatite B (cf. rappel sur les infections chroniques B et description détaillée du rationnel de l'étude, chapître IV.1).

1.3.2 c : Rappel sur le gène d'intérêt et la stratégie (cf paragraphe 1.2).
Le protocole s'inscrit dans l'approche générale du développement de la vaccination ADN pour l'immunothérapie des maladies infectieuses.

1.3.2 d : Les protéines d'intérêt sont des protéines d'enveloppe du virus de l'hépatite B. De nombreux travaux ont montré leur capacité à induire des anticorps neutralisants et une réponse cellulaire protectrice contre l'hépatite B. Des travaux plus récents ont démontré la faisabilité et l'intérêt de la vaccinothérapie spécifique du virus de l'hépatite B pour le contrôle de la multiplication virale chez les porteurs chroniques du virus B.

1.3.2 e : Type d'approche génétique

Il s'agit d'une approche indirecte visant à stimuler la réponse immunitaire au virus par l'injection intra-musculaire d'un vaccin à base d'ADN nu codant pour des protéines d'enveloppe du virus.

1.3.2 f : Il s'agit d'un vaccin ADN constitué d'un plasmide recombinant permettant l'expression des protéines d'enveloppe préS2/S sous le contrôle d'un promoteur ubiquitaire (CMV) (cf paragraphe 1.2).

1.3.2 g : La justification scientifique du choix de l'OGM est l'absence de toxicité maintenant décrite dans plusieurs études basées sur cette méthodologie. Elle est également basée sur la qualité des résultats préliminaires obtenus à la fois dans des modèles animaux et chez l'homme.

1.3.2 h : Quel est l'intérêt majeur que présente cette recherche ?

Le traitement de l'hépatite chronique B reste un traitement difficile dans un fort pourcentage de patients. L'importance des effets secondaires et le coût des traitements actuels justifient la poursuite de recherche visant à améliorer cette thérapeutique.

1.3.3. Etat de la recherche sur la maladie et sur son pronostic ; qualification de l'investigateur dans la pathologie concernée

Une mise au point détaillée sur l'hépatite chronique B est fournie dans le paragraphe IV 1.1. Schématiquement, les traitements antiviraux actuels permettent le contrôle de la multiplication virale dans environ 30 à 40% des cas. La poursuite d'une multiplication virale avec des signes importants d'activité histologique et de fibrose est un facteur de risque majeur pour la constitution d'une cirrhose avec ses complications sévères propres et en particulier le développement du cancer primitif du foie. L'équipe du Service d'Hépatologie de l'Hôpital Necker-Enfants Malades (Professeur Christian Bréchot, Professeur Stanislas Pol) a une expérience reconnue dans le traitement de l'infection par le virus de l'hépatite B. L'équipe du Professeur Tiollais, et en particulier le Docteur Marie-Louise Michel, à l'Institut Pasteur de Paris a une expérience reconnue internationalement sur la biologie moléculaire du virus de l'hépatite B et le développement des techniques d'immunisation à base d'ADN.

1.4 Recueil de l'information, suivi et audit

1.4.1 Modalités du recueil de l'information

Le recueil de l'information sera effectué par la combinaison d'un recueil d'informations cliniques et biologiques. Les informations cliniques seront recueillies dans un cahier de suivi spécifiquement mis au point pour cette étude. Ce recueil sera effectué de façon monocentrique pour ce premier essai de phase I (Service d'Hépatologie, Hôpital Necker-Enfants Malades, Paris).

Le recueil des données biologiques sera obtenu par :

- la réalisation de bilans hépatiques dans le Laboratoire de Biochimie A de l'Hôpital Necker-Enfants Malades,
- la constitution de sérothèques avant, pendant et après cet essai permettant l'évaluation de la multiplication virale B et de l'ensemble des marqueurs virologiques et sérologiques usuels de l'infection à VHB.

1.4.5 Quelles sont les procédures de suivi de l'étude envisagées ?

Nous proposons que, à coté du groupe d'investigateurs principaux un "comité scientifique" soit formé. Nous proposons la composition suivante pour ce comité scientifique :

- Responsables du protocole : Pr Bréchot, Pr Pol, Pr Tiollais, Dr Michel.
- Pr David Klatzmann (Hôpital Pitié Salpétrière, Paris).
- Pr Thomas Turz (Institut Gustave Roussy, Villejuif).
- Dr Jean-Loup Romet-Lemonne (Société IDM, Paris).
- Dr Françoise Degos (Service d'Hépatologie, Hôpital Beaujon, Clichy).
- Dr Renée Poupon (DR2 INSERM, INSERM U370).
- Dr Marie-Paule Kieny (DR2 INSERM, INSERM U74).
- Dr Jean-Luc Teillaud (DR2 INSERM, INSERM U255) représentant l'INSERM, promoteur du projet.

1.4.6 Des audits sont-ils prévus ? Si oui, par qui ?

Des audits pourraient être réalisés par le comité scientifique ad hoc.

1.5 Consentement éclairé

La note d'information aux patients est fournie en annexe 0.

1.6 Lieu de production et de contrôle, lieux d'administration et de contrôle.

1.6.1 Lieu de production

Le plasmide utilisé dans l'essai sera produit par l'unité de thérapie génique de la compagnie Qiagen (Max-Volmer Str. 4, Hilden, Allemagne) dans les locaux aux normes GMP de la compagnie Strathmann Biotech GmbH (Feodor Lynen Str. 5, Hannovre, Allemagne). Le contrôle qualité sera effectué par le personnel ad hoc de l'unité de thérapie génique de Qiagen (Hilden, Hannovre). L'ensemble des procédures est décrit dans l'annexe I.

1.6.2 Lieu d'administration et de contrôle

Service d'Hépatologie, Hôpital Necker-Enfants Malades, 149 rue de Sèvres, 75743 Paris Cédex 15.

Pharmacie Hospitalière (Professeur Singlas), Hôpital Necker-Enfants Malades, 149 rue de Sèvres, 75743 Paris Cédex 15.

I. RENSEIGNEMENTS RELATIFS A L'OGM : CONFINEMENT ET EVALUATION DES RISQUES DE DISSEMINATION

I.A Renseignements relatifs aux OGM impliqués aux différents stades de la production (classe de risque), et relatifs aux différents lieux impliqués : lieux de production, lieux d'administration, et laboratoires de contrôle (agrément).

I.A.1 Renseignements relatifs à l'OGM

I.A.1.1 Nature de l'OGM administré

Plasmide bactérien recombinant administré sous forme d'ADN nu par voie intramusculaire, permettant l'expression *in vivo* des protéines d'enveloppe du virus de l'hépatite B et l'induction d'une réponse immunitaire humorale et cellulaire spécifique de ces protéines.

I.A.1.2 Construction et production de l'OGM

a) Caractéristiques de l'organisme donneur et nature de l'insert

a.1. L'organisme donneur :

Génome du virus de l'hépatite B (VHB), virus humain du groupe des hepadnaviridae, sous type antigénique ayw. Le génome du VHB a été cloné, séquencé et l'étude de sa séquence nucléotidique a permis de définir les régions codant pour les protéines d'enveloppe du virus *(3)*. Séquence et carte du VHB en annexe II.

Classe de risque : III à l'état d'organisme, II pour les séquences mises en œuvre.

Origine subcellulaire de l'ADN utilisé : génome viral cloné dans des plasmides bactériens.

a.2. La nature de la séquence mise en oeuvre :

Séquences codantes : gènes d'enveloppe, région préS2 et gène S codant pour la protéine moyenne et pour la petite protéine d'enveloppe du VHB.
Protéines de structure du virus contenant des séquences antigèniques (sous type ayw).

Séquences non codantes et signaux d'expression : enhancer, signal de polyadénylation et signal d'export nucléaire des ARNm, signaux de sécrétion des protéines. Activité préférentielle de l'enhancer dans les cellules hépatiques. Les signaux de sécrétion fonctionnent de manière ubiquitaire dans les cellules animales.

La séquence utilisée est dérivée du plasmide PCP10 contenant deux copies du génome VHB clonées en tandem au site EcoRI *(4)*. Le fragment utilisé (2003 bp) est compris entre les sites SauI (nt 3165) et Bgl II (nt 1986) du génome VHB (voir carte en annexe II).

b) Pour chaque étape de la construction et de la production de l'OGM

b.1. Caractéristiques du micro-organisme récepteur impliqué

Souche de Escherichia coli DH5 alpha: supE44, delta lac U169 (phi 80 lacZdelta M15), hsdR17, recA1, endA1, gyrA96, thi-1, relA1.

Classe de risque I

Non pathogène pour l'homme, les animaux et les plantes

b.2. Systèmes vecteurs utilisés, association vecteur-séquence

Vecteur de clonage et d'expression pcDNA3 (Invitrogen, voir carte en annexe III). Le fragment utilisé (3556 nt) est compris entre le site BamH1 (nt 907) du polylinker et le site BssHII en 5' du gène de la néomycine (nt 2676). Ce fragment contient l'origine de réplication du plasmide (ColE1), le gène de résistance à l'ampicilline, les séquences enhancer-promoteur des gènes précoces du cytomégalovirus (CMV).

Le fragment SauI-Bgl II du VHB a été cloné entre les sites BamHI et BssHII du vecteur pcDNA3. Le plasmide obtenu a été appelé pCMVHB-S2.S (CNCM numéro I-1410) puis pCMV-S2.S (Michel et al., PNAS, 1995). (**Voir carte détaillée page suivante** et séquence en annexe III).

I.A.1.3 Classement évalué et proposé par le demandeur pour les différentes étapes de la construction et de la production de l'OGM :
Classe I, groupe 1 pour l'utilisation de fragments clonés bien caractérisés en systèmes bactériens d'expression.
Le point fondamental est le respect des normes GMP pour la production du plasmide destiné à être injecté chez l'homme. Ce plasmide sera produit par la société Qiagen, qui assurera un contrôle de qualité et de pureté (voir annexe I.1). Il sera fourni aux

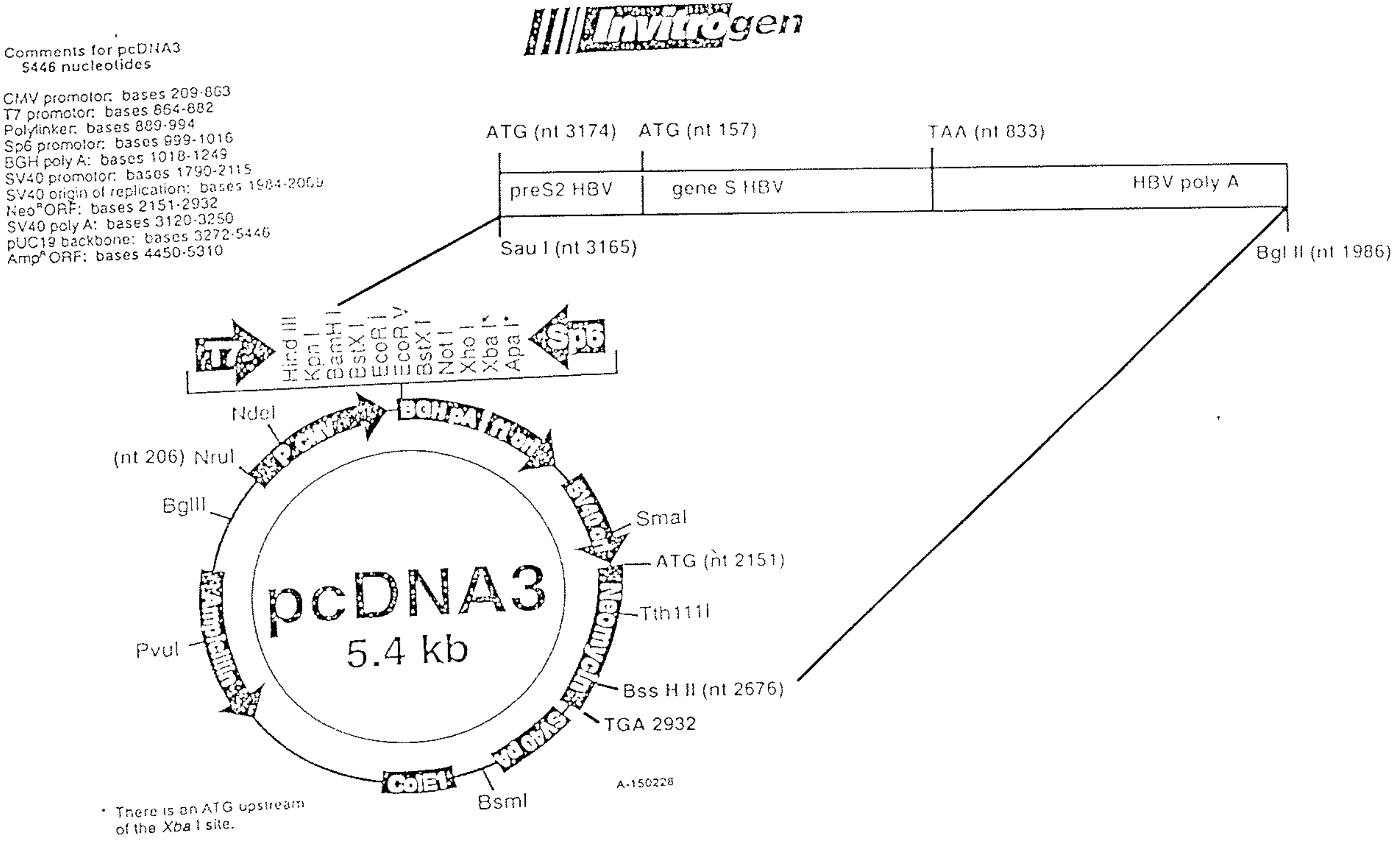

Vecteur pCMVHB-S2.S : le fragment Sau I - Bgl II de l'HBV
a été inséré entre les sites Bam HI et Bss H II du vecteur pcDNA3

médecins effectuant l'essai. L'ADN sera aliquoté en fioles de verre scellées au moment et sur le lieu de production.

I.A.2 Renseignements relatifs aux différents lieux

I.A.2.1 Renseignements relatifs au personnel

Réalisation de l'étude dans le Service d'Hépatologie de l'Hôpital Necker-Enfants Malades : Christian BRECHOT, PU-PH ; Stanislas POL, PU-PH ; Hervé ZYLBERBERG, CCA ; Hélène FONTAINE, CCA.

I.A.2.2 Renseignements relatifs aux conditions de confinement

Classe 1, Groupe I, Confinement L1, confinement de l'hospitalisation TL1 (**voir page suivante décision de la Commission de Génie Génétique**).

I.B. Renseignements relatifs aux interactions entre les OGM, l'environnement et l'entourage

Il n'y a pas de mesure particulière à prévoir concernant les interactions entre l'OGM envisagé, l'environnement et l'entourage dans la mesure où il s'agit d'une vaccination à ADN nu n'impliquant pas de vecteurs vivants.

Aucune incidence sur l'environnement n'est attendue (**voir page suivante l'avis favorable de la Commission du Génie Biomoléculaire du 17/12/99, référence : B/FR/99.10.01**).

L'importation et le transport seront réalisés conformément aux normes européennes en vigueur, le produit étant maintenu à – 20°C.

Le vaccin ADN sera stocké en permanence à –20°C.

L'ensemble des équipements nécessaires pour la réalisation et le suivi de l'étude (réfrigérateurs, congélateurs pour le maintien dans des conditions ad hoc des sérothèques, etc) sont déjà disponibles dans le Service d'Hépatologie de l'Hôpital Necker.

I.B.5 Sortie de l'essai des patients

Pas de mesure particulière de confinement nécessaire.

R E P U B L I Q U E   F R A N Ç A I S E

MINISTÈRE DE L'EDUCATION NATIONALE,
DE LA RECHERCHE ET DE LA TECHNOLOGIE

DIRECTION
DE LA RECHERCHE

Paris, le 27 Octobre 1999

Madame Sophie SEGOND
Agence Française de Sécurité
Sanitaire des Produits de Santé
Direction de l'Evaluation
143/145 Boulevard Anatole France
93200 SAINT DENIS

Objet Décision de classement

Madame,

Nous avons bien reçu la fiche de renseignements pour un essai clinique utilisant un organisme génétiquement modifié, portant le numéro TG 99.09.01., intitulé « **Vaccinothérapie spécifique par ADN nu au cours des hépatites chroniques B** » dont le promoteur est **l'INSERM** et l'investigateur coordonateur le Professeur Christian BRECHOT, et enregistré sous le n° **3472.**

Après examen par la Commission de Génie Génétique au cours de la séance du 21 Octobre 1999, le classement pour les manipulations de l'ADN nu telles que décrites dans le protocole de vaccinologie est :

Classe 1, Groupe I, Confinement L1, confinement d'hospitalisation TL1.

Professeur Jean-Pierre ZALTA
Président de la
Commission de Génie Génétique

1 rue Descartes, 75231 PARIS Cedex 05   Tel : 01 46 34 48 09   Fax : 01 46 34 49 97

COMMISSION DU GENIE BIOMOLECULAIRE

Paris, le 17 DEC. 1999

AVIS

La Commission du Génie Biomoléculaire réunie en séance, le 7 décembre 1999, sous la présidence du Professeur Marc FELLOUS, a procédé à l'examen du dossier déposé par **HOPITAL NECKER (investigateur-coordinateur), relatif "à un essai phase I de vaccinothérapie spécifique par ADN nu au cours des hépatites chroniques B comme traitement des hépatites chroniques dues au virus de l'hépatite B"**, enregistré sous la référence B/FR/99.10.01.

1. But de la dissémination

Le dossier est relatif à une demande d'autorisation pour un essai clinique visant à évaluer le pouvoir thérapeutique des réponses immunes induites contre l'antigène de surface de l'hépatite B chez des porteurs chroniques du virus injecté sous forme d'un plasmide d'expression ou d'ADN nu. Il s'agit d'un essai de phase I.

2. Caractéristisques de l'OGM disséminé

Le vecteur utilisé est un vecteur d'expression qui porte les gènes de résistance la néomycine et à l'ampicilline, l'origine de réplication (ColE1) et les séquences enhancer promoteur des gènes précoces du cytométagalovirus (CMV). Le plasmide recombinant pCMVHB-S2.S code pour deux protéines d'enveloppe du virus de l'hépatite B. Les deux régions codantes d'intérêt sont sous le contrôle des gènes précoces du cytomégalovirus. Le vaccin utilisé est constitué du plasmide recombinant produit dans des bactéries *E. coli* DH5 α.

3. Evaluation des risques pour la santé publique et l'environnement

Le plasmide bactérien recombinant est administré sous forme d'ADN nu par voie intramusculaire. Le plasmide permet l'expression in vivo des protéines d'enveloppe du virus de l'hépatite B et conduit à l'induction d'une réponse immunitaire humorale et cellulaire spécifique de ces protéines. Les tests conduits sur animaux avec ce plasmide n'ont pas révélé de réactions inflammatoires ou inattendues. Des études ont permis de disposer de plus de 6 années de recul sur le plasmide considéré ce qui constitue une garantie satisfaisante sur la sécurité de l'OGM.

II. RENSEIGNEMENTS PHARMACEUTIQUES ET BIOLOGIQUES

II.1 Brève présentation de la stratégie thérapeutique proposée

Il s'agit d'un protocole de vaccination thérapeutique à base d'ADN, utilisant un plasmide recombinant codant pour les protéines d'enveloppe PréS2/S et S, destiné à stimuler la réponse immune contre ces protéines virales chez des porteurs chroniques du virus de l'hépatite B.

II.2 Construction de l'OGM

II.2.1. Gène à transférer

1. La **séquence nucléotidique** du plasmide à injecter est fournie en Annexe III. Cette séquence a été déterminée au moment de la construction du plasmide et publiée dans le brevet N° PCT/FR 94/00 483 déposé le 27/04/1994. **Ce plasmide a été reséquencé** à partir d'un lot préclinique qui nous a été fourni par la société QIAGEN. Cinq mutations mineures de la séquence intervenant dans des régions non codantes du plasmide ont été notées (voir les conclusions du document « séquençage du plasmide pCMV-S2.S d1» en Annexe III). Ce lot de plasmide a été utilisé pour l'immunisation de souris et de singes macaques Rhésus.

2. La séquence des gènes codant pour les protéines d'enveloppe du VHB n'a pas été modifiée (cf. Annexe II, séquence et carte VHB).

II.2.2 Stratégie adoptée pour le transfert de gène

Utilisation d'un acide nucléique non viral nu, administré *in vivo* par voie intramusculaire.

II.2.2.3. Utilisation d'un acide nucléique non viral nu

1. La construction

- Le vecteur de clonage et d'expression : pcDNA3 (Invitrogen, voir carte en Annexe III). Ce vecteur contient, en aval du promoteur des gènes précoces du cytomégalovirus (pCMV, nt 209 -nt 863), un polylinker (nt 889 à nt 994). Le fragment du VHB a été cloné entre le site BamH I du polylinker et le site BssHII en

3' du gène de la néomycine (nt 2676). Le gène codant pour la néomycine situé entre les nt 2151 et 2932 est donc en grande partie éliminé. Le fragment restant de pcDNA3 (3676 nt) contient le signal de polyadénylation dérivé de SV 40, l'origine de réplication du plasmide (ColE1), le gène de résistance à l'ampicilline, les séquences enhancer-promoteur des gènes précoces du cytomégalovirus (pCMV). Le signal de polyadénylation dérivé du gène de l'hormone de croissance bovine (BGH pA), le promoteur des gènes précoces et l'origine de réplication dérivés du virus SV 40 ainsi que la plupart du gène codant pour la résistance à la néomycine ont été éliminés.

- L'insert : le fragment SauI-Bgl II du VHB a été cloné entre les sites BamHI et BssHII du vecteur pcDNA3. La séquence utilisée est dérivée du plasmide pCP10 contenant deux copies du génome du VHB clonées en tandem au site EcoRI (*ref.* 4 et en Annexe II). Le fragment utilisé (2003 bp) est compris entre les sites SauI (nt 3165) et Bgl II (nt 1986) du génome VHB (voir carte en Annexe II).
- Le plasmide obtenu a été appelé pCMVHB-S2.S (CNCM numéro I-1410) puis pCMV-S2.S (*ref.* 1 et en annexe III), brevet d'invention n° FR 93 12659 délivré à l'Institut Pasteur, PCT/FR 94/00 483 déposé le 27/04/1994.
- **Un schéma de l'insertion du fragment HBV dans le vecteur pcDNA3 est fourni p 10 bis** et en complément de l'Annexe III.

2. Caractéristiques du micro-organisme producteur

a) Souche de Escherichia coli DH5 alpha :

supE44, delta lac U169 (phi 80 lacZdelta M15), hsdR17, recA1, endA1, gyrA96, thi-1, relA1.

Cette souche transformée par le plasmide pCMVHB-S2.S a été déposée à la Collection Nationale de Cultures de Micro-organismes (CNCM) sous le numéro I-1410 le 22/04/1994.

3. Méthode d'obtention du clone d'intérêt

La production et les contrôles qualité relatifs au plasmide seront effectués par le personnel ad hoc de l'unité de thérapie génique de la société QIAGEN GmbH (Max-Volmer Strasse 4, 40724 HILDEN, Allemagne) dans les locaux de la société Strathmann Biotech GmbH (Feodor-Lynen Str. 5, Hannovre, Allemagne).

Il sera établi :

- une première banque dite « RCB pour Research Cell Bank », constituée dans des conditions GLP, à partir de bactéries E. Coli DH5 alpha transformées par le plasmide parental pCMV-S2.S fourni par le laboratoire d'origine. Les ampoules de bactéries congelées en glycérol constituant cette banque seront pour une partie (20 ampoules) stockées à -20°C ou à une température inférieure et pour une autre partie utilisées pour établir :
- une banque primaire « MCB ou Master Cell Bank » contenant des bactéries viables E. Coli DH5 alpha transformées par le plasmide parental, issues de la RCB et cultivées en conditions GMP. Ces bactéries diluées en glycérol seront stockées en ampoules scellées à une température inférieure à -80°C (300 ampoules). Elles répondront aux spécifications énoncées dans le document « EXHIBIT III » fourni par QIAGEN (Annexe I.1).
- une banque de travail « MWCB, Manufacturer Working Cell Bank » contenant des bactéries viables E. Coli DH5 alpha transformées par le plasmide parental, issues de la MCB et cultivées en conditions GMP. Ces bactéries diluées en glycérol seront stockées en ampoules scellées à une température inférieure à -80°C (300 ampoules). Elles répondront aux spécifications énoncées dans le document « EXHIBIT IV » fourni par QIAGEN (Annexe I.1).

Les bactéries transformées par le plasmide pCMV-S2.S seront cultivées dans un milieu ne contenant pas d'ampicilline, permettant une croissance optimale des bactéries, selon des conditions déterminées par le producteur dans un essai pilote. Voir le document en annexe I.4 « technologie de fermentation pour la production de plasmide » et l'annexe I.3 pour les matériels et milieux de culture.

4. Analyse du plasmide dans le système hôte :

Une séquence complète du plasmide sera effectuée sur le plasmide issu de la MCB et sur le produit fini et comparée à la séquence de départ.

La MCB et la MWCB seront caractérisées selon les spécifications et avec les méthodes décrites dans les documents « EXHIBITS III et IV » (Annexe I.1). Celles-ci incluent une carte de restriction du plasmide pour garantir que le plasmide contient les éléments attendus.

II.3 PRODUCTION ET CONTRÔLES DE ROUTINE

II.3.1 Description du procédé de production des lots de Principe Actif vrac purifié

II.3.1.1. Schéma

Le principe actif est un ADN plasmidique purifié à partir de bactéries en culture.
Un schéma de la technologie de fermentation des bactéries et de la purification de l'ADN est fourni en Annexe I.4 ("Fermentation technology for plasmid production" et "Quality assurance et quality control in plasmid DNA manufacturing").

II.3.1.2. Description du procédé de production

L'ADN sera purifié par chromatographie sur colonne échangeuse d'ions (technique Qiagen). Au cours de cette procédure de purification l'ADN n'entrera en contact avec aucun agent toxique tel que le phénol, le chloroforme ou le bromure d'éthidium. La production sera réalisée conformément aux normes cGMP en accord avec les recommandations US 21 CFR. La technologie de purification proposée a été approuvée par les autorités concernées pour des essais cliniques en Europe (Medicine Control Agency MCA en Angleterre) et aux USA (FDA, drug master file type II: BB-MF 6224 et BB-MF 7882), voir annexe I.2.

Voir les documents fournis par la société QIAGEN en annexe I.4 :

- Plasmid DNA for clinical Phase I and II studies;
- Issues of large scale plasmid DNA manufacturing;
- Production of plasmid DNA in industrial quantities according to cGMP guidelines.

II.3.1.3. Nature et qualité des produits utilisés au cours des phases de production

Voir les fiches (contrôle de qualité) fournies par QIAGEN en annexe I.4.

II.3.2. Contrôles

1- Le plasmide sera séquencé au niveau de la banque primaire ou MCB et sa séquence sera comparée à celle du plasmide de départ. Une carte de restriction sera établie.

2- A partir d'une fraction de la banque MWCB, une fermentation et un lot pilote d'ADN sera préparé en vue de tests in vitro et de tests d'efficacité in vivo chez l'animal (voir : « EXHIBIT VI » en annexe I.1).
3- L'intégrité du plasmide sera contrôlée par séquençage du produit fini, son homogénéité par migration en gel d'agarose et son identité par digestion au moyen d'enzymes de restriction appropriés.
La pureté de l'ADN sera contrôlée par HPLC, par recherche d'ADN génomique de E.coli, d'ADN simple brin et linéaire, d'ARN, d'endotoxines et de protéines bactériennes.
Une recherche de viabilité bactérienne sera effectuée (Bioburden assay).
Pour les spécifications des contrôles et les méthodes utilisées, voir: « EXHIBIT II» en annexe I.1.
Le produit fini sera accompagné d'un dossier qui fera état des différents procédés de purification et des résultats des contrôles.

II.3.3 Contrôle de qualité du produit fini

II.3.3.1 : Spécifications et contrôles de qualité des excipients et véhicules intervenant dans la formulation galénique.
Sans objet.

II.3.3.2 : Forme pharmaceutique et contrôles du produit fini

II.3.3.2.1 : produit fini prêt à l'emploi

L'ADN produit à partir de la MWCB dans des conditions GMP sera resuspendu à la concentration d'un milligramme par ml dans une solution apyrogène de chlorure de sodium à 9 pour mille.
Le produit fini sera conditionné, de manière stérile sous hotte à flux laminaire, dans des fioles de verre stériles fermées par des bouchons en bromobutyl fixés par un capuchon d'aluminium. Chaque fiole étiquetée contiendra 1 ml soit 1 mg d'ADN pCMV-S2.S (voir annexe I.3).
Les contrôles de qualité du produit fini ont été décrits au paragraphe II.3.2.

II.4. UTILISATION DU PRODUIT

II.4.1. Traitement in vivo

La suspension de plasmide sera administrée à la seringue par voie intramusculaire.

II.5. STOCKAGE

II.5.1 Stockage

Les conditions de transport et de stockage des monodoses seront les suivantes :

- envoi sous carboglace par frêt express par la société QIAGEN ;
- stockage par la pharmacie de l'hôpital Necker à -20°C dans des congélateurs prévus à cet effet.

Une étude de stabilité du plasmide en fin de production sera effectuée dans les conditions proposées de stockage par analyse en gel d'agarose des différentes formes du plasmide.

Cette stabilité sera vérifiée tous les six mois par analyse en gel d'agarose.

Le produit sera libéré pour une utilisation de deux ans après la fabrication du lot ; la validité du produit sera prolongée sur la base de l'étude de stabilité mentionnée ci-dessus.

Après décongélation le produit ne sera pas recongelé.

II.5.2 Méthode de traçabilité des lots cliniques

La traçabilité sera réalisée grâce à la collaboration entre la société Qiagen, le Service d'Hépatologie et la pharmacie de l'Hôpital Necker-Enfants Malades. Le produit fourni par Qiagen sera précisément répertorié et cette information sera disponible en permanence à la pharmacie de l'Hôpital Necker.

II.5.3 Retours des échantillons non utilisés et modalités de destruction

Les échantillons non utilisés seront détruits. Leur destruction ne posera pas de problème particulier.

III. ELEMENTS DE PHARMACO-TOXICOLOGIE

III.1. DONNEES DE PHARMACO-TOXICOLOGIE SUR LE GENE D'INTERET

III.1.1 Données pharmacologiques sur le gène d'intérêt

III.1.1.1 Connaissance du gène

Région préS2 et gène S du virus VHB codant pour deux protéines d'enveloppe du virus au moyen de deux codons d'initiation en phase. Ces gènes ont été bien caractérisés depuis le clonage du virus (1979) et peuvent être exprimés dans tout type de cellules eucaryotes.

III.1.1.2 Connaissance de la protéine exprimée

Les deux protéines codées par les gènes préS2 et S existent sous forme glycosylée et non glycosylée dans l'enveloppe du VHB. Leur localisation dans les cellules eucaryotes est cytoplasmique. Il s'agit de protéines qui s'auto-assemblent avec des lipides d'origine membranaire pour former des pseudoparticules virales vides qui sont sécrétées. Elles ont été produites sous forme recombinante à partir de cellules eucaryotes et sont la base du vaccin recombinant anti-hépatite B « GenHevac B ».

III.1. 2. Données toxicologiques sur le gène d'intérêt

Ce sont des protéines de l'enveloppe virale dotées d'un fort pouvoir antigénique. Elles sont inductrices d'anticorps neutralisants lors de la vaccination (anticorps anti-HBs) et de lymphocytes T auxiliaires et cytotoxiques lors de l'infection naturelle.

III.2. DONNEES DE PHARMACO-TOXICOLOGIE SUR LES AUTRES GENES

Le plasmide contient un gène bactérien de résistance à l'ampicilline.

III.3. DONNEES DE PHARMACO-TOXICOLOGIE SUR L'OGM

III.3.1. Données pharmacologiques sur l'OGM

L'administration se fera par voie intramusculaire à raison de trois injections d'un milligramme d'ADN à deux mois d'intervalle. Une injection de rappel sera effectuée six mois plus tard (soit au dixième mois).

Biodistribution : il a été montré chez la souris et le lapin que, lors de l'administration intramusculaire, l'ADN circule initialement par voie sanguine vers tous les tissus mais est retrouvé majoritairement dans le tissu musculaire au site d'injection où il peut persister pendant huit semaines environ *(5)*.
L'expression du gène d'intérêt est sous contrôle du promoteur CMV et peut donc avoir lieu dans tout type de tissus. Chez la souris, l'expression des protéines codées par le gène d'intérêt a été documentée dans le muscle et la protéine a été retrouvée dans le sang circulant où elle est probablement secrétée *(6)*.

III.3.2. Données toxicologiques sur l'OGM

Au cours des essais de tolérance chez l'animal et des premiers essais cliniques chez l'homme, il n'a pas été mis en évidence d'anticorps anti-ADN, ni de pathologie auto-immune, ni de toxicité après des injections multiples d'ADN nu *(2, 5, 7, 8, 9)*.

III.4. DONNEES DE PHARMACO-TOXICOLOGIE DU PRODUIT ADMINISTRE

III.4.1 Données de pharmacologie du produit administré

III.4.1.1 Efficacité du transfert et de l'expression du gène

1. Evaluation moléculaire

Des études réalisées chez la souris au moyen de méthodes de fractionnement d'ADN et d'amplification par PCR des séquences plasmidiques, ont montré que l'ADN plasmidique persiste dans le muscle à raison d'environ 3 à 30 copies/150000 génomes 30 à 60 jours après l'injection. Il n'a pas été possible de savoir si ces séquences correspondaient à des séquences intégrées ou à de l'ADN lié de manière covalente à l'ADN génomique *(10)*. En tout état de cause, si l'ADN plasmidique était intégré, le taux calculé de mutations serait 3000 fois moins que le taux de mutations spontanées dans un génome de mammifère *(10)*.

2. Evaluation biologique

Les protéines exprimées à partir du gène d'intérêt porté par le plasmide ont été visualisées par immunofluorescence dans le tissu musculaire et par ELISA dans le sérum des souris injectées. Les anticorps spécifiques de ces protéines sont détectés chez la souris à niveau constant pendant plus de 18 mois, suggèrant une stimulation à long terme du système immunitaire par l'antigène produit. Chez le chimpanzé, en absence d'anticorps détectables un an après immunisation, la réponse anticorps peut être rappelée par injection de protéine recombinante *(11)* ou au cours d'une épreuve

virulente *(12)* suggérant qu'une réponse mémoire a été induite par une production constante de l'antigène.

3. Modifications phénotypiques induites dans les cellules cibles

Les cellules cibles sont les cellules du tissu musculaire qui sont dans un état post-mitotique. L'absence de division cellulaire de ces cellules limite les risques d'intégration au moment de la mitose.

4. Etudes sur animaux

Cette partie est reprise et détaillée dans le chapitre IV. 1

- Plusieurs études précliniques ont été réalisées chez l'animal et ont montré que l'injection du plasmide pCMV-S2.S stimule la production d'anticorps anti-enveloppe (anticorps anti-HBs) dont on sait qu'ils sont protecteurs chez l'homme et le chimpanzé. Les titres anticorps obtenus sont supérieurs à 10mUI/ml chez la souris après une injection et chez le primate après deux injections (Rhésus macaque et chimpanzé). Ce titre en anticorps est défini comme conférant la protection chez l'homme. L'injection du plasmide stimule également l'immunité cellulaire. Chez la souris et le macaque Rhésus, des lymphocytes T cytotoxiques spécifiques des protéines d'enveloppe du VHB ont été mis en évidence.

  Chez le macaque et chez le chimpanzé, il n'a pas été montré d'effet secondaire de ce type d'immunisation, ni au point d'injection, ni au niveau des paramètres biologiques (paramètres hématologiques de routine et enzymes du foie).
- Dans deux modèles animaux des porteurs chroniques du virus, d'une part des souris transgéniques pour l'antigène de surface du VHB et d'autre part des canards chroniquement infectés par le virus correspondant du groupe hepadna (DHBV), il a été montré que la vaccination génétique avec un plasmide codant pour les protéines d'enveloppe permet de lever la tolérance à l'antigène HBs et d'induire une réponse humorale et cellulaire spécifique de cet antigène. De plus, chez la souris, les lymphocytes T induits par la vaccination génétique contrôlent l'expression des gènes viraux et la réplication virale par un mécanisme non cytopathique impliquant l'interféron. Ces expériences suggèrent que la vaccination génétique pourrait stimuler une réponse immunitaire défaillante chez les porteurs chroniques, et ainsi permettre à l'individu de contrôler son infection. Chez ces animaux, il n'a pas été noté d'atteinte hépatique en réponse à la vaccination génétique, en particulier on ne note pas d'élévation des transaminases ni de modifications histologiques du foie (pas de nécrose ni d'infiltration lymphocytaire).

III.4.1.2 Justification de la dose et de la voie d'administration chez l'animal

Les doses choisies ont été extrapolées des expériences publiées récemment chez le primate et chez l'homme (cf. III.4.1.1.2 et IV.1.2).
L'intervalle de temps entre deux administrations nous parait justifié par les résultats obtenus antérieurement et par la nécessité d'une évaluation à la fois de la toxicité et de l'effet éventuel sur la multiplication virale après chaque injection.

III.4.2 : Pharmacocinétique du produit administré

Les données disponibles actuellement ne permettent pas de décrire réellement une pharmacocinétique précise du produit.

III.4.3 : Données toxicologiques du produit administré

Ces études seront confiées à la société Phoenix (L'Arbresle, France). Les paragraphes suivants en décrivent les protocoles de façon résumée.

III.4.3.1 : Toxicité aiguë

Une étude toxicologique de contrôle sera réalisée sur le produit fini GMP :

Toxicité en dose unique par voie intraveineuse chez le rat.

- Espèce : Rat OFA.SD.
- Age à l'initiation de l'étude : 6 semaines
- Nombre d'animaux dans l'étude : 30 (15 males and 15 femelles).

- Schéma expérimental

Numéro du groupe	Nom du groupe	Niveau de dose (mg/kg)	Nombre d'animaux	
			Males	Femelles
1	Controle	0	5	5
2	Dose faible	0.1	5	5
3	Dose forte	1.0	5	5

Tous les animaux seront sacrifiés au jour 15.

- Traitement: une seule administration (bolus) par voie intraveineuse au jour 1.
- Mortalité/morbidité et signes cliniques: 15 minutes après l'injection de l'article à tester, puis à 1, 2 et 4 heures, et chaque jour pendant la durée de l'étude.

- Poids corporels : immédiatement avant le traitement (jour 1), les jours 8 et 15 et en cas de mort à partir du jour 1.
- Nécropsie : échantillonage du foie et recherche des anomalies macroscopiques
- Histopathologie du foie en cas d'anomalie macroscopique.
- Conditions de BPL.

III.4.3.2 : Toxicité répétée

Une étude toxicologique de contrôle sera réalisée sur le produit fini GMP :

Toxicité sur 8 semaines chez le rat par voie intramusculaire.

- Espèce: Rat OFA.SD.
- Age à l'initiation de l'étude: 6 semaines
- Nombre d'animaux dans l'étude: 30 (15 males and 15 femelles).

Schéma expérimental

Numéro du groupe	Nom du groupe	Niveau de dose (mg/kg)	Nombre d'animaux Males	Femelles
1	Controle	0	5	5
2	Dose faible	0.05	5	5
3	Dose forte	0.5	5	5

Tous les animaux seront sacrifiés le jour suivant la dernière administration (jour 44).

- Traitement: injection intramusculaire de l'article à tester une fois toutes les deux semaines, avec un maximum de 4 injections (days 1, 15, 29 and 43).
- Mortalité/morbidité et signes cliniques: 15 minutes après l'injection de l'article à tester, puis à 1, 2 et 4 heure, et deux fois par jour pendant la période d'étude.
- Tolérance locale: examens des sites d'injection chaque jour. Analyse histopathologique des muscles injectés à la fin de l'étude.
- Poids corporels: immédiatement avant le traitement, et une fois par semaine pendant la durée de l'étude.
- Consommation de nourriture: chaque jour.

- Biologie : Avant la première administration, et à la fin de l'étude
  - Hématologie
  - Chimie clinique sérique
- Nécropsie : liste complète des organes/tissus
- Histopathologie
- Conditions de BPL.

III.4.3.3 : Etudes à long terme

Les études dont on dispose actuellement ne permettent pas de répondre aux critères "long terme".

III.4.3.4 : Etudes du potentiel de dissémination du gène

Sans objet dans cette étude (avis favorable de la Commission du Génie Biomoléculaire du 17/12/99 référencé : B/FR/99.10.01).

III.4.3.5 : Toxicologie des fonctions de reproduction

Aucun argument en faveur d'un effet sur les fonctions de reproduction. Il est de toute façon prévu de demander l'utilisation d'une contraception efficace aux patients.

III.4.3.6 : Evaluation des risques de mutagénèse

Aucun argument en faveur d'une mutagénèse insertionnelle dans l'ensemble de la littérature disponible sur ce sujet.

III.4.3.7 : Potentiels cancérogènes

Aucun argument en fonction de l'ensemble des données disponibles.

III.4.3.8 : Données immunologiques

L'induction d'une réponse immune à la fois humorale et cellulaire spécifique est un des avantages majeurs de la vaccination ADN. Il n'a pas été mis en évidence d'anticorps anti-ADN dans les études réalisées chez le primate et l'homme.

III.4.3.9 : Autres effets toxicologiques : *Sans objet dans cette étude.*

III.4.3.10 : Tolérance locale

Aucun problème de tolérance locale rapporté dans les protocoles déjà réalisés.

IV –ESSAI CLINIQUE

IV.1 RATIONNEL

IV.1.1 Etat actuel des connaissances/thérapeutiques alternatives

L'infection par le virus de l'hépatite B et ses modalités thérapeutiques actuelles
Pour revue générale, voir : *(32, 33, 34)*.

Histoire naturelle de l'infection par le virus de l'hépatite B (VHB)
L'infection par le virus de l'hépatite B (VHB) est largement répandue dans le monde : on estime à plus de 300 millions le nombre de porteurs chroniques du virus de l'hépatite B sur le globe avec schématiquement des régions à forte prévalence de l'antigène HBs (Afrique, Asie du Sud Est) , des régions à prévalence intermédiaire (Italie, Afrique du Nord, Espagne du Sud, Grèce, Japon) et des régions de prévalence faible (Europe du nord et Etats-Unis d'Amérique) où respectivement 5 à 10%, 2 à 5% et moins de 2% de la population générale est porteuse chronique de l'antigène HBs. La transmission du VHB est principalement parentérale, sexuelle et materno-fétale. Dans les zones de forte endémie la contamination est de type verticale (Asie) ou horizontale en période périnatale (Afrique).
Le VHB est un virus à ADN enveloppé. La physiopathogénie de l'hépatite B est essentiellement immunomédiée. La réponse immunitaire, en particulier cellulaire induit la nécrose et l'apoptose hépatocytaire par reconnaissance des antigènes viraux exprimés sur la membrane des hépatocytes. Ces mécanismes pathogéniques, où l'interaction hôte/virus a un rôle central, rendent compte de la diversité de présentations de cette hépatite. La réaction inflammatoire, avec la libération de cytokines (TNF-α en particulier), joue un rôle essentiel dans le développement de la fibrose hépatique et la prolifération cellulaire.
L'histoire naturelle de l'infection virale B peut être résumée de la façon suivante. A la suite d'une hépatite aiguë, symptomatique dans 10-30% des cas (dont 1 % sous une forme fulminante), la guérison survient spontanément chez environ 95 % des adultes immunocompétents avec disparition de l'AgHBs et apparition des anticorps antiHBs et antiHBc. Le problème principal de l'infection virale B, est celui du portage chronique de l'antigène HBs. Il survient dans environ 5 % des cas chez l'adulte immunocompétent, mais plus fréquemment chez les enfants infectés tôt dans la vie

(40-90 %) ou chez les immunodéprimés (30 à 100%). En l'absence de mesures prophylactiques, plus de 80 % des enfants nés de mère ayant une infection active par le VHB développeront en effet une infection chronique. A côté de l'âge à la contamination et des situations d'immunosuppression patentes, les facteurs influençant le passage à la chronicité sont immunogénétiques. Il a été suggéré que le passage à la chronicité était associé à l'absence d'HLA DR2 et à la présence d'HLA DR7. Des études comparatives ont montré que l'absence de passage à la chronicité était associée à une forte réponse immune cellulaire dirigée contre les antigènes HBc et HBe avec un profil de type Th1. Le fait que le passage à la chronicité soit moins fréquent en cas d'hépatite aiguë symptomatique renforce cette hypothèse.

L'infection chronique par le VHB est classiquement définie par le portage de l'AgHBs 6 mois après l'hépatite aiguë. Elle est caractérisée par son polymorphisme de présentation allant des porteurs sains de l'antigène HBs (1/3 des infectés chroniques) aux sujets présentant une hépatite chronique associée à une réplication virale qui peut diminuer au cours du temps. La multiplication virale responsable de l'hépatite chronique (par la reconnaissance des antigènes viraux exprimés à la surface des hépatocytes détruits par les cellules du système immunitaire), les arrêts spontanés de multiplication virale souvent accompagnés d'une exacerbation de l'hépatite et d'éventuels épisodes de réactivation rendent compte de l'évolution possible vers la cirrhose (20% des sujets). La cirrhose expose par elle-même au risque de carcinome hépatocellulaire, risque renforcé par l'action directe de certaines protéines virales et l'insertion de l'ADN VHB dans l'ADN cellulaire (32).

La multiplication virale est définie par la détection dans le sérum de l'ADN du VHB, associée dans 85 % des cas à la positivité de l'antigène HBe ; dans 15 % des cas, les anticorps anti-HBe sont présents témoignant, le plus souvent, d'une infection par un virus mutant (mutation dans le gène préC). La biopsie hépatique, outre la confirmation de l'hépatite chronique, permettra de préciser l'activité de l'hépatite par l'établissement d'un score incluant des index semi-quantitatifs de nécrose péri-portale et intralobulaire, d'inflammation et de fibrose (score de Knodell ou score Métavir). Elle en établira sa sévérité (cirrhose ou non). Elle permettra de détecter dans les hépatocytes l'antigène HBc par immunohistochimie dont la présence témoigne de la multiplication virale.

La cirrhose est un événement crucial dans l'histoire naturelle de l'hépatite chronique B car les complications propres (hypertension portale et insuffisance

1990 et 1994. Cette tendance est encore plus nette dans la tranche des 6-9 ans où cette même incidence passe de 0,52 chez les enfants nés entre 1974 et 1984 à 0,13 pour 100 000 enfants nés entre 1984 et 1986 *(35)*.

Traitements de l'hépatite chronique B *(50)*

Les risques mentionnés liés à l'infection chronique par le VHB soulignent d'une part l'intérêt indiscutable de la vaccination prophylactique, traitement le plus efficace en terme de santé publique et d'économie de santé, et d'autre part l'importance des prises en charge diagnostiques et thérapeutiques chez les sujets ayant une hépatite chronique. Le caractère immunomédié de l'hépatite chronique B explique les stratégies thérapeutiques potentiellement antivirales ou immunomodulatrices, trouvant leur point d'orgue dans l'interféron-α qui combine ces 2 propriétés.

Le but des traitements antiviraux est l'éradication de la multiplication virale afin d'éviter la constitution d'une cirrhose et par là même diminuer les risques de cancer. Les indications classiques du traitement antiviral sont actuellement restreintes à une infection virale B responsable d'une hépatite chronique histologiquement prouvée avec une multiplication virale détectable.

L'interféron-α

La posologie traditionnelle de l'INF-α est de 2,5 MU/m^2 (soit 5 à 6 MU 3 fois par semaine) pour des durées de 4 à 6 mois. Au-delà de cette période le gain de réponse efficace ne justifie pas le coût financier et les effets secondaires. L'interféron- α est administré par auto-injection sous-cutanée, 3 fois par semaine.

Les résultats des différentes études contrôlées sont tous en faveur du traitement avec environ 40% de négativation de l'antigène HBe et de l'ADN du VHB sérique et près de 10% de négativation de l'antigène HBs ; chez les sujets non traités, ces taux sont respectivement de l'ordre de 10% et de 0%. L'arrêt de la multiplication virale s'accompagne d'une amélioration des index histologiques d'activité et d'une disparition complète de l'ADN viral sérique, recherché par amplification génomique (PCR), chez la moitié des patients 5 ans après un arrêt de multiplication virale attesté par les méthodes conventionnelles d'hybridation en phase aqueuse *(36)*.

Le traitement des infections liées à un mutant préC pose plus de problème : ces infections, souvent associées à une maladie histologiquement plus sévère que celles liées au virus sauvage et à une multiplication virale modérée, sont rarement contrôlées par les traitements antiviraux standards du fait des rechutes fréquentes à

l'arrêt du traitement ; cependant, des traitements prolongés seraient durablement efficaces chez bon nombre de patients.

Pour le VHB, un certain nombre de facteurs prédictifs d'une réponse positive à l'interféron ont été identifiés : une ancienneté d'infection inférieure à 2 ans, le caractère symptomatique de l'hépatite aiguë initiale, une réplication virale faible ou modérée (moins de 200 pg/ml d'ADN sérique du VHB), une hypertransaminasémie supérieure à 3 fois la valeur supérieure de la normale. D'autres facteurs sont prédictifs d'une mauvaise réponse au traitement : une contamination péri-natale ou une immunosuppression, notamment dans le cadre d'une infection par le VIH.

L'arrêt de la multiplication virale au stade d'hépatite chronique permettrait d'éviter l'évolution vers la cirrhose et l'apparition de ses complications en cas de cirrhose. Le traitement peut être prescrit chez des patients ayant une cirrhose compensée ou non. En cas de cirrhose décompensée, la posologie sera habituellement la moitié de la posologie standard : dans ce type d'indication, certains patients ayant favorablement répondu au traitement ont pu éviter une transplantation hépatique ; les patients traités ayant une cirrhose décompensée seront très régulièrement suivis du fait du risque de dégradation de leur fonction hépatique en cas d'efficacité antivirale accompagnée d'une exacerbation de leur hépatite *(46, 47)*.

La diminution de l'activité de l'hépatopathie diminue logiquement le risque de carcinome hépatocellulaire mais ce point n'a pas jusqu'à présent été formellement démontré. Cependant, les risques liés à l'intégration de l'ADN viral et à la persistance d'expression de certaines protéines virales persistent.

Peu d'études sont disponibles sur l'analyse des bénéfices à long terme du traitement par l'interféron. Dans une étude non randomisée comparant 103 patients ayant une hépatite chronique B traités par interféron et suivis 5 ans en moyenne à 53 patients non traités *(36)*, les taux cumulés de disparition des AgHBe et AgHBs étaient, dans les 2 groupes, de 56% et 11,6% versus 28,1% et 0% respectivement. Parmi les patients traités 15,5 % ont présenté une complication liée à la cirrhose et 5,8% sont décédés de cause hépatique. Seule une complication (et aucun décès) n'était observée parmi les patients ayant négativé l'antigène HBe sous traitement. Le facteur principal associé à une survie sans complication était la disparition de l'antigène HBe. Il apparaît donc que, du fait d'une diminution d'évolution vers la cirrhose et ses complications, l'efficacité d'un traitement par interféron est associée à un bénéfice clinique à long terme.

Les autres thérapeutiques antivirales: les nouveaux analogues nucléotidiques (50)
Le développement de la recherche pharmaceutique pour le traitement de l'infection VIH a permis des progrès révolutionnaires pour le traitement des hépatites virales. Des analogues nucléotidiques ont été récemment développés. Si leur utilisation est actuellement restreinte à des protocoles thérapeutiques ou à des autorisations temporaires d'utilisation, leur efficacité doit être soulignée. Si certains, telle la fialuridine, ont été rapidement abandonnés du fait d'une toxicité mitochondriale inacceptable car responsable d'une stéatose microvésiculaire mortelle, la lamivudine apparait comme le traitement le plus prometteur.

La lamivudine (ou 3TC) est un inhibiteur nucléotidique de la transcriptase réverse du virus de l'immunodéficience humaine (VIH) ; elle est aussi un puissant inhibiteur de la réplication du virus de l'hépatite B (VHB) par sa capacité d'inhibition des activités ADN- et ARN-dépendantes de l'ADN polymérase des hépadnavirus. Contrairement à la plupart des autres analogues nucléotidiques inhibiteurs de la transcriptase réverse, la lamivudine semble dénuée de toute toxicité mitochondriale.
Cet effet antiviral sur le VHB, initialement décrit *in vitro* et *in vivo* chez l'animal, a été confirmé en clinique humaine, à la fois chez des patients immunodéprimés (VIH) ou non immunodéprimés.
Dans une étude récente, la négativation de l'ADN VHB survenait dans 75 à 100% des cas et dans un délai de 1 à 2 mois selon la dose utilisée (100% pour une dose de 100 ou 300mg/24h). Une reprise de la multiplication virale était observée dans 80% des cas à l'arrêt du traitement. Les effets secondaires étaient nuls ou minimes avec en particulier l'absence de toxicité rénale.
Bien que les essais thérapeutiques n'en soient qu'à un stade préliminaire, on retiendra : 1. la très bonne tolérance du produit (des augmentations asymptomatiques des enzymes musculaires ou pancréatiques sont observées chez environ un tiers des patients) ; 2. l'exceptionnelle efficacité pour des posologies supérieures ou égales à 100 mg par jour avec une amélioration histologique des activités nécrotico-inflammatoires hépatiques ; 3. la nécessité de traitements prolongés d'au moins 12 mois qui limitent le risque de rechute post-thérapeutique à environ 15% ; 4. la fréquente induction de résistance génotypique par mutations ponctuelles dans le gène codant pour la polymérase virale chez environ 15% des sujets immunocompétents traités et 27% des transplantés hépatiques. La résistance se caractérise par un échappement avec réaugmentation des transaminases et

réapparition d'une multiplication virale détectable qui avait initialement disparu. Quoique son intérêt soit indiscutable, les risques élevés d'échappement en cas de poursuite du traitement ou de réactivation à l'arrêt en limitent l'utilisation large en monothérapie en dehors des situations de "sauvetage".

En résumé, les traitements antiviraux par l'interféron sont marqués par une efficacité seulement partielle ; les nouveaux analogues nucléotidiques apportent un avantage indiscutable aux patients ayant des hépatopathies sévères (et notamment aux candidats à la transplantation hépatique) mais restent associés à des taux très élevés de réactivation virale à l'arrêt des traitements ou à des risques de sélection de mutants d'échappement. Il est logique dans ces conditions de développer des stratégies thérapeutiques immuno-modulatrices. Si les immuno-suppressions, notamment par la corticothérapie, ont montré leur effet délétère sur l'histoire naturelle de l'infection virale B, d'autres stratégies incluant le transfert passif d'immunité ou l'utilisation d'immuno-stimulations non spécifiques par des facteurs de croissance ou des dérivés thymiques sont en cours d'évaluation.

Vaccinothérapie spécifique au cours de l'hépatite B

Un certain nombre d'arguments fondamentaux et cliniques suggèrent que la vaccination au cours d'une infection chronique puisse apporter un bénéfice pour contrôler l'infection. En effet, au cours des infections chroniques, la réponse immunologique dirigée contre les antigènes du pathogène est sous-optimale mais existe, permettant d'espérer en cas de modification de la présentation des antigènes (par exemple par la vaccination) d'optimiser cette réponse immunologique. Plusieurs essais de vaccinothérapie ont été réalisés au cours d'infections virales (Herpès, VIH), parasitaires, ou bactériennes (tuberculose) avec des résultats encourageants.
Pour ce qui concerne l'infection par le virus de l'hépatite B, les lymphocytes B des porteurs chroniques de l'antigène HBs sont capables, *in vitro,* d'excréter après stimulation spécifique des anticorps anti-HBs neutralisants. De plus, la vaccination de souris transgéniques, exprimant de façon constitutive l'antigène HBs ou le génome complet du VHB, entraine une diminution de la production de l'antigène HBs, voire une négativation de la production de l'ADN du VHB *(37, 38)*. Des études pilotes ont été réalisées chez l'homme à partir de ces données expérimentales. Ces études pilotes ont suggéré une réelle efficacité puisque environ 30 % des sujets ayant une infection chronique par le VHB, associée à une hépatite chronique, ont eu au

cours du suivi, une négativation de l'ADN viral B après vaccination spécifique (3 à 6 injections) *(48, 49)*. Ces études pilotes ont ouvert la voie au développement d'une première étude multicentrique coordonnée par le service d'hépatologie de l'Hôpital Necker.

Son but était d'évaluer cette efficacité potentielle dans une étude contrôlée *(42)*. Elle incluait 110 sujets AgHBs+ ayant un ADN du VHB sérique détectable et une hépatite chronique histologiquement prouvée, dont 10 % avec cirrhose, randomisés dans un groupe témoin ne recevant pas de traitement pendant 12 mois alors que les 2 autres recevaient 6 injections d'un vaccin préS2/S (Genhevac B -PMC) ou d'un vaccin S (Recombivax -MSD), (3 injections de 20μg d'AgHBs à un mois d'intervalle puis 3 de 20 μg d'AgHBs à 3 mois d'intervalle). Le critère d'efficacité était la négativation de l'ADN du VHB à 6 et 12 mois par le test Digene. L'induction de réponses prolifératives lymphocytaires spécifiques était mesurée avant et après vaccination.

Plusieurs éléments permettaient d'affirmer la réalité d'un effet positif de la vaccination (sans que des différences d'efficacité soient mises en évidence entre les vaccins PréS2/S et S) :

La négativation du test de détection de l'ADN VHB sérique était observée au 6ème mois chez 13/84 sujets vaccinés (15.5%) et seulement 1/37 témoins (2.7%) (p=0.04).

Une séroconversion HBe-antiHBe n'était observée au 6ème mois que chez les sujets vaccinés.

Des anticorps anti-PréS2 étaient détectés chez les sujets vaccinés par le vaccin PréS2/S.

Un travail expérimental a permis de clairement montrer l'induction par la vaccination d'une prolifération lymphocytaire T, spécifique des antigènes vaccinaux (PréS2/S ou S) et médiée par des cellules CD4 (TH1), chez ces sujets porteurs chroniques du VHB *(39)*.

Cette étude donne donc pour la première fois, une base rationnelle au concept de vaccinothérapie spécifique au cours de l'infection VHB. Elle montre cependant clairement les limitations de cette approche : en effet, du fait d'un taux élevé (environ 25%) d'arrêt spontané de multiplication du VHB dans le groupe contrôle à 12 mois, une différence significative de négativation de l'ADN VHB sérique et de séroconversion HBe-antiHBe n'était plus observée après un an de suivi. De plus, la négativation du test de détection de l'AgHBs n'était observée chez aucun patient alors qu'elle est de 10% environ chez les sujets traités efficacement par interféron-α.

Ces données soulignent donc :

- *l'efficacité potentielle de cette approche,*
- *la nécessité d'élaborer des stratégies d'immunisation plus efficaces.*

IV.1.2 Le produit a-t-il déjà fait l'objet d'essais cliniques ?

La vaccination ADN/VHB n'a pas été utilisée jusqu'à présent en Europe.
Des essais cliniques de vaccination à base d'ADN ont été déjà réalisés chez l'homme dans des essais de phase I ou II. A titre préventif des vecteurs codant pour des protéines du *plasmodium falciparum* ont été injectés à des sujets naïfs (28 volontaires) et ont stimulé une réponse cytotoxique spécifique de plusieurs épitopes du parasite et ceci dans plusieurs contextes HLA *(7)*. Les doses d'ADN injectées par voie intramusculaire allaient de 20 à 2500 µg et des réponses cytotoxiques ont été obtenues après seulement deux injections de 20 microgrammes d'ADN. L'injection de 500 ou 2500 microgrammes d'ADN induisait malgré tout une réponse significativement meilleure. A titre thérapeutique deux essais ont été réalisés chez des patients chroniquement infectés par le virus VIH. Le but de ces approches thérapeutiques vaccinales était de rappeler une réponse préexistante, mais faible, et d'induire une réponse cellulaire cytotoxique de longue durée visant à contrôler la réplication virale.
Dans un essai réalisé en Suède *(9)* , on a pu observer chez 8/9 des patients ayant reçu l'ADN vaccin (3 injections de 100 microgrammes par voie IM), une augmentation de la fréquence des précurseurs de cellules T cytotoxiques spécifiques du VIH ainsi qu'une réponse proliférative transitoire. Dans un autre essai réalisé aux USA *(8)* les 15 patients ont reçu 3 doses d'ADN vaccin de 30, 100 ou 300 microgrammes par voie IM en présence d'un facilitateur pour la capture de l'ADN, la bipuvacaine. Dans cet essai, il n'a pas été noté de réactions locales ou systémiques, ni d'anormalités dans les analyses de laboratoire courantes. En particulier, aucun anticorps anti-ADN, ni élévation des enzymes musculaires n'ont pu être mis en évidence dans le sérum des patients vaccinés.

IV.1.3 Principaux résultats en terme d'efficacité de transfert et d'efficacité thérapeutique :

Vaccination spécifique par immunisation à base d'ADN, résultats concernant le VHB dans des modèles animaux

Les résultats encourageants de la vaccinothérapie spécifique utilisant des protéines recombinantes d'enveloppe du VHB soulignent l'intérêt de cette approche chez les porteurs chroniques du virus. Néanmoins, les résultats sont encore insuffisants et doivent être améliorés. Une nouvelle méthodologie de vaccination a récemment vu le jour et est basée sur l'injection intramusculaire ou intradermique d'ADN codant pour des antigènes d'intérêt *(13; 14)*. Elle a été appelée vaccination génétique et consiste à induire une réponse immunitaire à une protéine, exprimée *in vivo* directement à partir d'un ADN plasmidique nu *(15, 16)*. Ce nouveau mode de vaccination est particulièrement efficace puisqu'il permet d'obtenir à la fois une réponse humorale et une réponse cellulaire spécifiques du pathogène étudié et d'induire une protection contre une épreuve virulente dans un modèle animal lorsque celui-ci existe. Cette efficacité particulière est liée d'une part à la néosynthèse *in vivo* des antigènes à partir des ADN plasmidiques injectés, ce qui permet leur dégradation intracellulaire par le protéasome et leur présentation sous forme de peptides antigéniques associés et présentés en surface par les molécules de classe I du CMH. Ceci conduit à l'induction d'une forte réponse cellulaire de type cytotoxique capable de tuer les cellules infectées. D'autre part, la production d'antigènes par les cellules injectées ou ayant capturé l'ADN permet d'obtenir une réponse humorale spécifique et une réponse cellulaire T auxiliaire restreinte par les molécules de classe II du CMH. La forte réponse Th1 obtenue par l'immunisation génétique est à mettre au compte de la présence dans les plasmides bactériens de motifs d'ADN activateurs de l'immunité innée et appelés motifs CpG. Les vaccins à base d'ADN contiennent ainsi leur propre adjuvant stimulant la voie Th1 de l'immunité et agissant en synergie avec la réponse spécifique à l'antigène.

Dans le modèle de l'hépatite B, la protection est médiée par les anticorps spécifiques de l'enveloppe virale (Ac anti-HBs). On admet généralement que lors de l'infection aiguë par le VHB, la réponse humorale et la réponse cellulaire sont toutes deux requises pour obtenir la disparition du virus et la guérison. Lors de la résolution spontanée de l'infection, ces réponses sont fortes, polyclonales et dirigées contre

toutes les protéines du virus. Par contre ces réponses sont de spécificité plus restreinte, beaucoup plus faibles et transitoires dans le cas des infections chroniques. La vaccination génétique apparaît comme un outil potentiel pour stimuler efficacement les réponses humorales et cellulaires nécessaires au contrôle de l'infection virale.

Des essais de vaccination prophylactique ou thérapeutique ont été réalisés dans différents modèles animaux. Un modèle de vaccin génétique anti-hépatite B, basé sur l'utilisation de vecteurs plasmidiques codant pour les trois protéines d'enveloppe du virus a été mis au point. Ces vecteurs, injectés en une seule dose par voie intramusculaire chez la souris permettent la synthèse de l'AgHBs par les cellules du tissu musculaire. Cet antigène est détecté transitoirement dans le sérum des souris à des taux faibles *(6)*. Différents vecteurs ont été construits permettant l'expression de l'une ou l'autre des protéines d'enveloppe. Leur injection chez la souris induit des anticorps qui sont dirigés contre tous les déterminants de l'enveloppe virale (préS1, préS2 et S) *(17)*. Les conditions affectant le transfert de gènes ont été étudiées chez la souris, le rat et le lapin, et il a été montré que le prétraitement du muscle permettait d'obtenir une réponse humorale plus précoce et 10 fois plus intense que l'injection dans le muscle mature *(18)*. Les anticorps obtenus sont stables pendant plus de 18 mois et la réponse anticorps peut être rappelée aussi bien par injection d'ADN que par l'injection de protéine recombinante purifiée *(19)*. La réponse humorale obtenue mime celle que l'on peut observer lors d'une infection virale aussi bien pour la cinétique que pour la spécificité des anticorps induits *(1)*.

La supériorité de l'immunisation à base d'ADN par rapport à l'injection de protéine a été montrée dans plusieurs haplotypes de souris où la réponse à l'AgHBs est restreinte par les molécules H-2. L'injection d'ADN codant pour les protéines d'enveloppe VHB chez des souris non-répondeuses à l'injection de protéine HBs permet de court-circuiter la non-réponse et d'induire une réponse anticorps précoce. Ceci pourrait être particulièrement important chez les individus qui répondent mal au vaccin que cette non-réponse soit dûe à leur HLA ou à d'autres facteurs connus pour compromettre la réponse au vaccin *(20)*. La vaccination à base d'ADN, de par la néosynthèse des antigènes, permet également l'induction d'une réponse cytotoxique spécifique des antigènes exprimés. Chez la souris BALB/c ($H2^d$) une réponse cytotoxique intense a été obtenue même après une seule injection de 10 microgrammes d'ADN dans du muscle mature. Cette réponse, spécifique des antigènes viraux, est détectée dès une semaine après l'injection et persiste pendant au

moins 12 semaines *(29)*. Ces résultats, obtenus chez la souris, ont été validés par plusieurs groupes *(22, 23, 24, 31)* et confirmés dans d'autres modèles animaux d'infection par les hépadnavirus. Dans le modèle du virus de la marmotte (WHV), une réponse cellulaire de type proliférative a pu être mise en évidence chez cet animal après vaccination génétique avec un plasmide codant pour l'enveloppe, conduisant à la protection lors d'une épreuve virulente par le virus WHV *(25)*. Dans le modèle du virus du canard DHBV, des anticorps spécifiques des différents domaines de l'enveloppe ont été obtenus et permettent de neutraliser le virus *in vitro* et de protéger les animaux d'une infection *(26)*.

Ce nouveau mode d'immunisation a été validé chez le primate en vue d'un éventuel passage à l'homme. Des macaques Rhésus immunisés par des vecteurs codant pour les protéines d'enveloppe du VHB développent une réponse anticorps anti-HBs à un niveau compatible avec une protection chez l'homme. De plus, chez les animaux immunisés une fréquence élevée de cellules T cytotoxiques spécifiques du VHB a été détectée. D'autre part, dans ce modèle primate il a été montré que l'injection d'ADN pouvait permettre de rappeler une immunité ancienne résultant d'injections d'antigènes recombinants classiques *(21)*. Dans le modèle du chimpanzé, des expériences d'immunisation menées avec les mêmes vecteurs ont montré que les titres anticorps obtenus étaient largement compatibles avec une protection chez l'homme. Ces anticorps pouvaient par ailleurs être rappelés par une injection à distance de protéine recombinante indiquant qu'une mémoire B avait été induite *(11)*. L'infection par le VHB étant souvent contractée par voie périnatale, des chimpanzés nouveau-nés ont été également immunisés à la naissance par les mêmes vecteurs. Ces animaux ont été ensuite éprouvés par voie intraveineuse un mois après la vaccination. Une protection a été observée chez ces animaux en l'absence de réponse anticorps détectable. Ces animaux ont été malgré tout infectés à bas bruit comme le montre la détection d'ADN viral par PCR, mais l'infection a été contrôlée, vraisemblablement par la réponse cytotoxique induite par la vaccination *(12)*.

Pour aborder le problème du traitement des porteurs chroniques du virus VHB, des souris transgéniques pour l'AgHBs ont été utilisées comme modèle animal. Ces souris expriment les trois protéines d'enveloppe du VHB dans le foie uniquement et sécrètent l'AgHBs dans le sérum à des taux élevés. Ces souris ne développent aucune pathologie du foie et représentent un modèle pour les porteurs chroniques asymptomatiques sans réplication virale. Il a été montré, chez ces souris, que la

vaccination génétique permet de lever la tolérance à l'AgHBs, d'induire sa clairance sérique et de contrôler dans le foie l'expression des gènes viraux *(27)*. L'analyse de la réponse immunitaire induite chez les souris transgéniques après vaccination, a mis en évidence le rôle des cellules T dans le contrôle *in vivo* de l'expression des gènes viraux au niveau du foie. Des expériences de transferts adoptifs de sous-populations lymphocytaires montrent que cette régulation s'effectue aussi bien par les cellules CD4+ que par les cellules CD8+ spécifiquement activées par l'immunisation génétique. L'immunisation de souris transgéniques déficientes pour le récepteur à l'interféron-γ suggère que ces cellules agissent par le biais de la sécrétion de cytokines de type Th1 (interféron-γ et TNF-α). De plus, cette régulation s'effectue en l'absence de toute attaque lytique du foie *(28)*. Cette nouvelle méthodologie de vaccination, en induisant des anticorps neutralisants et une réponse T capable de contrôler les gènes viraux représente donc une approche thérapeutique potentielle pour le traitement des porteurs chroniques du virus VHB. La vaccination de canards chroniquement infectés, au moyen d'ADN codant pour les protéines d'enveloppe, confirme ces résultats puisque l'on note chez certains animaux infectés une diminution voire une élimination des formes réplicatives du virus DHBV*(26)*.

IV.1.4 Principaux effets secondaires observés

Deux types d'effets secondaires doivent être envisagés :

Les effets secondaires éventuels "généraux" de la vaccination VHB :

En dehors des effets locaux (douleur au point d'injection) ou généraux (fièvre), l'actualité a récemment mis l'accent sur les risques éventuels de la vaccination contre le VHB, à laquelle ont été attribuées des atteintes neurologiques démyélinisantes centrales (sclérose en plaques, myélite transverse) et des pathologies générales auto-immunes. Ces cas ont été décrits dans une population qui de façon très majoritaire, était génétiquement prédisposée à la SEP (sexe féminin, groupe HLA DR2, antécédents familiaux de SEP), voire chez des patients ayant déjà connu des poussées préalables *(40)*. En France, au 31 mars 1998, 271 atteintes démyélinisantes centrales, 160 pathologies auto-immunes et 107 atteintes hématologiques secondaires à une vaccination étaient dénombrées. Pour ces complications, le nombre de cas notifiés à la pharmacovigilance, ramené au nombre de personnes vaccinées (25 à 27 millions de sujets), ne dépassent pas la prévalence connue dans la population non vaccinée. En particulier, dans plusieurs études conduites récemment, le nombre de scléroses en

plaques observé dans la population vaccinée n'est pas significativement accru par rapport à celui de la population non vaccinée *(41)*. L'incidence spontanée de la sclérose en plaques est de l'ordre de 1 à 3 pour 100 000 par an en France, soit entre 500 et 1 000 nouveaux cas ; la coïncidence entre l'apparition d'une sclérose en plaques ou d'une myélite transverse à la suite d'une vaccination massive contre l'hépatite B ne constitue donc pas en soi la preuve d'une relation causale entre la vaccination et les symptomes observés. C'est pourquoi, à ce jour, les autorités n'ont pas retenu de lien causal, d'autant plus que dans la plupart des pays européens (et aux USA) où des programmes de vaccination des nourrissons et/ou des adolescents ont été systématiquement proposés, aucun pays n'a signalé de recrudescence d'évènements indésirables de type neurologique associés à la vaccination.

Ces difficultés sont particulièrement illustrées par le suivi de l'étude controlée de vaccination contre le virus de l'hépatite B que nous avons menée chez les porteurs chroniques. En effet nous avons observé un cas de neuropathie démyélinisante et celui-ci est survenu chez un sujet du groupe **témoin** !!!

L'arbitrage de la commission d'A.M.M. en 1997 a cependant demandé à ce que soit ajoutée aux mentions légales du vaccin une contre-indication chez les patients atteints de SEP ou ayant des antécédents familiaux de manifestation neurologique démyélinisante. Le lien entre stimulation immunitaire vaccinale et poussées de sclérose en plaques est, par ailleurs, connu de longue date. D'autres effets indésirables ont été décrits de manière exceptionnelle tels l'apparition de cryoglobulinémie, de périartérite noueuse, d'uvéite, de vascularite, d'acrodermatite. Le caractère immunomédié de ces réactions pourrait plaider en faveur de l'origine vaccinale de ces manifestations, cependant on ne peut exclure, de part leur caractère sporadique, de simples coïncidences. La rareté de ces cas chez l'adulte, le lien spécifique non prouvé avec la vaccination contre le VHB et l'absence de cas décrits chez l'enfant doivent faire poursuivre les campagnes de vaccination, en particulier des populations à risque et des enfants nouveaux-nés.

Les effets secondaires éventuels spécifiques de la vaccination chez les porteurs chroniques du VHB

La vaccination d'un sujet porteur chronique du VHB pourrait théoriquement conduire à deux types de complication :

- Aggravation majeure de l'insuffisance hépatocellulaire par nécrose des hépatocytes détruits par la réponse immune au vaccin. Une élévation des AST et des

ALT est fréquemment observée chez les sujets chez qui un arrêt ou une diminution significative de multiplication virale B est obtenue. Cette élévation est également observée, en dehors de ces protocoles de vaccinothérapie, chez les sujets qui arrêtent spontanément la multiplication virale ou qui arrêtent cette multiplication au cours d'un traitement par l'interféron-α. Dans le protocole de vaccination que nous avons conduit cette cytolyse a été parfois importante (jusqu'à 10 fois la normale) mais ne s'est jamais associée à une aggravation significative de l'insuffisance hépatocellulaire. Ce risque théorique conduira cependant à exclure d'un protocole de vaccination (de même qu'actuellement pour tout protocole de traitement par interféron-α ou autre antiviral) des patients présentant une cirrhose "décompensée".

- Développement de maladies liées à la formation de complexes immuns antigène HBs-antiHBs secondaires à la vaccination. La recherche de complexes immuns s'est révélée négative dans notre essai thérapeutique et nous n'avons observé aucun cas de glomérulo-néphrite ou vascularite.

CONCLUSIONS :

1. Bien que l'efficacité des traitements antiviraux ait été améliorée au cours des dix dernières années, elle reste nettement insuffisante :

- *Efficacité globale antivirale de l'interféron α de l'ordre de 30 à 40 %.*
- *Réactivation fréquente de la multiplication virale sous traitement par analogue nucléotidique liée à l'émergence de mutations du génome viral.*

2. Des résultats encourageants ont été obtenus à partir de deux approches complémentaires:

- *Chez l'homme: protocole de vaccination par le vaccin HBV "classique" chez des porteurs chroniques du virus B avec une réduction de multiplication virale de l'ordre de 30 à 40%, comparable en fait à celle obtenue par l'interféron α, et la démonstration de l'induction d'une rupture de la tolérance immune et d'une prolifération lymphocytaire T spécifique des antigènes viraux*
- *Dans des modèles animaux: résultat très encourageant chez la souris, le canard et le primate de la vaccination ADN/HBV.*

3. Aucun effet secondaire n'a été observé au cours des protocoles de vaccination HBV (vaccin "classique") et des premières études de vaccin ADN.

IV.2 Objectifs

IV.2.1 Objectif principal :

Evaluer la tolérance à un traitement basé sur une vaccination ADN pour l'induction d'une réponse immune contre les protéines PréS2/S et S du VHB chez des sujets chroniquement infectés par le virus de l'hépatite B. Cet essai sera le prélude à des essais cliniques visant à apprécier l'efficacité de cette stratégie thérapeutique sur la multiplication virale, les tests biologiques et les lésions histologiques de patients atteints d'hépatite chronique actives dues au virus B.

IV.2.2 Objectifs secondaires :

- Analyser la réponse immune avant et après vaccination ADN.
- Rechercher si l'induction de cette réponse pourrait favoriser la sélection de certaines mutations du génome viral.

IV.3 Patients

IV.3.1 Nombre de patients dans l'essai

- Nombre de patients prévus dans l'essai : 10.
- Il ne s'agit pas d'un essai comparatif.
- Modalités du recrutement : ces patients sont suivis dans le service d'hépatologie de l'hôpital Necker Enfants Malades à Paris. La sélection sera faite en fonction des critères d'inclusion détaillés ci-dessous.

IV.3.2 Critères d'inclusions/non inclusions

IV.3.2.1 Critères d'inclusion :

1. Caractéristiques générales des patients :

Les patients inclus seront chroniquement infectés par le virus de l'hépatite B (antigène HBs positif depuis au moins un an). Une biopsie hépatique aura permis d'affirmer le diagnostic d'hépatique chronique active avec ou sans cirrhose. Ces patients auront été traités avec les meilleurs traitements actuellement disponibles :

interféron-α et lamivudine. Les bilans virologiques biologiques et histologiques récents montreront :

- la persistance de la multiplication virale à plusieurs examens répétés (2 sur 3 mois au minimum),
- l'existence d'une inflammation : activité sérique des transaminases supérieure à deux fois la normale, hépatite chronique active (biopsie hépatique datant de moins de trois ans montrant, suivant la classification Métavir, des scores de fibrose et d'activité d'au moins 2).

Ces caractéristiques justifieront en effet l'essai thérapeutique chez ces patients : il s'agit de maladies actives, à évolution potentiellement sévère, ayant résisté aux meilleurs traitements disponibles.

2. Sur quels éléments (cliniques, paracliniques) du diagnostic de la maladie les patients sont-ils sélectionnés ?

- Age : 18 à 60 ans
- Portage chronique de l'antigène HBs, associé ou non à l'antigène HBe.
- Détection dans le sérum de l'ADN du virus de l'hépatite B dont la quantification sera assurée par la technique Murex et/ou bDNA Chiron.
- Hépatite chronique prouvée histologiquement, avec ou sans cirrhose, scores de fibrose et d'activité de 2 à 4 (Métavir).

3. Formes cliniques recrutées : sur quels éléments sont-elles identifiés ? : cf. ci-dessus

4. Bilan immunologique : dans le cadre des objectifs secondaires du projet un bilan immunologique sera réalisé avant et après vaccination (cf. description du protocole) le bilan immunologique standard comprendra également la recherche à titre systématique d'anticorps anti-tissus avant et après vaccination.

5. Bilan vaccinal : cf bilan immunologique et virologique.

6. Bilan virologique : antigène HBs, antigène HBe, ADN du virus de l'hépatite B dans le sérum (Hybridation standard : test Murex ou Chiron).

7. Traitement antérieur ou en cours obligatoire : les patients seront sélectionnés sur un traitement antérieur inefficace par interféron-α et lamivudine.

IV.3.2.2. Critères de non inclusion :

1. Formes cliniques non incluses :

- Groupe HLA DR2.
- Cirrhoses décompensées (ictère, ascite ou encéphalopathie, taux de Quick < 60%).

2. Pathologies associées exclues :

- Infection associée par le virus de l'immunodéficience humaine, le virus de l'hépatite delta ou le virus de l'hépatite C.
- Toxicomanie intraveineuse active.
- Alcoolisme chronique.

3. Traitements antérieurs ou en cours ne permettant pas l'inclusion.

- Traitement immunosuppresseur en cours.

4. Risques de sortie de l'essai :

Pas de risque particulier créé par le protocole.

IV.4. Traitements :

IV.4.1 Traitement étudié

- Posologie proposée : le vaccin ADN sera utilisé en injections d'1 mg d'ADN.
- Voie et modalités d'administration : la voie intramusculaire sera utilisée. L'ADN sera administré en solution saline isotonique sous un volume total d'1 ml, réparti pour moitié entre les deltoïdes droit et gauche.

IV.4.2 Traitements systématiquement associés :

Aucun traitement ne sera associé à la vaccination ADN.

Une contraception efficace sera requise.

IV.4.3. Traitements non autorisés

Aucun traitement ne sera particulièrement contre-indiqué par la vaccination ADN (en dehors d'autres thérapeutiques antivirales spécifiques du VHB).

IV.5 Critères d'évaluation

IV.5.1. Critère d'évaluation principal de l'essai

- Quel est le critère d'évaluation principal de l'essai ? Le critère principal est la tolérance locale et générale aux injections : tolérance clinique et évolution des tests biologiques hépatiques.

- Ce critère est-il validé pour l'indication étudiée ? La recherche d'une aggravation de l'insuffisance hépatique est un critère validé de tolérance au cours de différents traitements antiviraux et évalué au cours de tout essai thérapeutique pour le VHB.

3-4-5 : Cette évaluation clinique et biologique sera réalisée mensuellement jusqu'au douzième mois, puis à M15, M18, M21 et M22.

IV.5.2 Critères d'évaluation secondaires de l'essai

- Evolution de la multiplication du virus de l'hépatite B : dosage de l'ADN viral (techniques d'ADN branché, Chiron), dosage des antigènes HBe et HBs. Recherche des anticorps anti HBs et anti HBe, recherche d'anticorps anti PréS2 (imposant un suivi d'un an après la dernière injection de vaccin). Les prélèvements seront réalisés à : M-2, M0, M2, M4, M6, M8, M10, M12, M15, M18 et M21.
- Evaluation de la réponse immune avant et après injection d'ADN. Cette évaluation sera réalisée avant l'injection du vaccin (M-2, M0) et un mois après chaque injection de vaccin (M1, M3, M5 et M11) et à M21.

IV.5.3 Recueil des données

Le recueil des données sera effectué de façon mensuelle jusqu'au douzième mois, puis à M15, M18, M21 et M22.

IV.5.4. Analyses statistiques

Il n'y aura pas d'analyse statistique dans cette première étude de tolérance.

IV.5.5. Biologie spécifique

Les examens spécifiques biologiques comprendront :

- les bilans hépatiques,
- une analyse de la multiplication virale telle que décrite dans les paragraphes précédents,
- une analyse de la réponse immune, (tests de prolifération lymphocytaire : *ref. 39)*. Activation de lymphocytes T (test ELISPOT).

Ces bilans seront réalisés comme décrits au chapitre IV.5.2.

IV.5.6. Questions spécifiques à l'évaluation de la tolérance

IV.5.6.1 Effets indésirables "attendus" liés aux produits évalués ?

Comme cela est décrit dans l'introduction à la vaccination ADN des effets indésirables ne sont pas particulièrement attendus dans ce protocole. L'évaluation de ces effets sera basée :

- Sur l'examen clinique : analyse de la tolérance locale et générale ;
- Sur des bilans biologiques hépatiques pratiqués mensuellement.

IV.5.6.2. Effets indésirables attendus liés aux traitements associés :

Sans objet dans ce protocole.

IV.5.6.3. Effets indésirables attendus liés à la maladie

- Comme cela est décrit dans l'introduction, on peut théoriquement envisager une aggravation de l'insuffisance hépatocellulaire provoquée par la rupture de tolérance et l'induction d'une réponse immune vis à vis des hépatocytes infectés. Ce type d'effet secondaire a été observé au cours du traitement par interféron-α. Une élévation des transaminases a été également observée lors des premiers protocoles de vaccination basés sur des vaccins standards contre le virus de l'hépatite B chez des porteurs chroniques (comme décrits dans l'introduction). Nous n'avons cependant jamais observé d'insuffisance hépatique sévère dans ces conditions. De même, dans une étude récente, basée sur l'injection d'un lipopeptide inducteur de réponses cytotoxiques anti-capside VHB, il n'a pas été observé d'effet délétère *(30)*.

Le protocole précise cependant que des patients atteints de cirrhose avec insuffisance hépatique importante (cf paragraphe IV.3.2.2) ne seront pas inclus.

2-3. Durée du suivi et fréquence des évaluations : évaluation mensuelle pendant les douze premiers mois de l'essai et à M15, M18, M21 et M22. Un suivi régulier (tous les trois mois) sera proposé aux patients dans le cadre du suivi normal de leur hépatite virale.

4. Mesures correctrices envisagées : une cytolyse supérieure à 5 fois la valeur normale et/ou une aggravation de l'insuffisance hépatocellulaire imposeraient une surveillance hebdomadaire (clinique et biologie hépatique) voire, en cas d'aggravation importante de l'insuffisance hépatique (taux de Quick < 50 %), une surveillance en milieu hospitalier.

IV.5.6.4.: Si l'essai prévoit une escalade de dose quels sont les critères de changement de doses ?

Sans objet.

IV.5.6.5. Quels sont les critères d'arrêt de traitement pour un patient

Le critère d'arrêt du traitement serait essentiellement une aggravation nette de l'insuffisance hépatocellulaire (taux de Quick inférieur à 50 %) et/ou une cytolyse supérieure à 10 fois la valeur normale.

IV.6 Déroulement de l'essai

IV.6.1. Résumé du protocole

Le protocole est basé sur l'utilisation d'un vaccin ADN permettant d'induire une réponse immune contre le virus de l'hépatite B.

Il comprend trois injections d'1 mg (1 ml), réalisées à deux mois d'intervalle par voie intramusculaire dans les régions deltoïdes droite et gauche, suivies d'une injection de rappel à 10 mois.

Le critère principal d'évaluation est la tolérance clinique et biologique à la vaccination.

Les critères secondaires sont l'effet sur la multiplication virale et la réponse immune spécifique du VHB.

Le suivi des patients est basé sur des consultations permettant un examen clinique et un bilan hépatique standard (voir calendrier individuel de suivi p 47).

L'inclusion des patients sera séquentielle : 2 patients seront inititialement inclus, puis quatre patients après un délai de surveillance de deux mois, les derniers patients étant inclus après une nouvelle période de surveillance de deux mois (voir calendrier de l'étude p 47).

Il n'y a pas d'hospitalisation envisagée.

IV.6.2. Sortie de l'essai :

Les critères de sortie éventuelle de l'essai seraient une aggravation de l'insuffisance hépatocellulaire (taux de Quick inférieur à 50%) et/ou une cytolyse supérieure à 10 fois la valeur normale.

Il n'y a pas de mesure ou précaution particulière à prendre pour les patients qui souhaiteraient sortir de l'essai

IV.7. Suivi à court et à long terme de l'essai

- Proposition de calendrier de soumission à l'Agence Française de Sécurité SAnitaire des Produits de Santé de rapports d'étude à intervalles réguliers :
  Nous proposons de soumettre un premier rapport quatre mois après la dernière injection de vaccin du dernier groupe (soit dix huit mois après l'initiation des vaccinations).

- Proposition d'un système de suivi à long terme des patients inclus dans l'essai :
  Les patients inclus dans l'essai sont régulièrement suivis dans le service d'Hépatologie de l'hôpital Necker-Enfants Malades.
  Un suivi tous les trois mois sera proposé permettant une évaluation clinique, biologique et virologique dans le cadre du suivi normal de leur hépatite virale.

Un comité de suivi de ce protocole est proposé : il incluera les personnalités suivantes:

- Responsables du protocole : Pr Bréchot, Pr Pol, Pr Tiollais, Dr Michel.
- Pr David Klatzmann (Hôpital Pitié Salpétrière Paris).
- Pr Thomas Turz, Institut Gustave Roussy, Villejuif.
- Dr Jean-Loup Romet-Lemonne (Société IDM, Paris).
- Dr Françoise Degos, Service d'Hépatologie, Hôpital Beaujon, Clichy.
- Dr Renée Poupon (DR2 INSERM, INSERM U370).
- Dr Marie-Paule Kieny (DR2 INSERM, INSERM U74).
- Dr Jean-Luc Teillaud (DR2 INSERM, INSERM U255) représentant l'INSERM, promoteur du projet.

CALENDRIER DE L'ETUDE

	M-2	M0	M2	M4	M6	M8	M22	M24	M26
Groupe 1 : 2 patients	Pré-incl.	Incl.					Sortie		
Groupe 2 : 4 patients		Pré-incl.	Incl.					Sortie	
Groupe 3 : 4 patients			Pré-incl.	Incl.					Sortie

Inclusion groupe 1 : M0 – Sortie d'étude : M22
Inclusion groupe 2 : M2 – Sortie d'étude : M24
Inclusion groupe 3 : M4 – Sortie d'étude : M26

Durée totale de la recherche : 28 mois (2 mois de pré-inclusion + 22 mois de traitement/suivi + 4 mois de décalage), soit environ 2 ans et quatre mois.

CALENDRIER INDIVIDUEL DE SUIVI

	M-2	M0	M1	M2	M3	M4	M5	M6	M7	M8	M9	M10	M11	M12	M15	M18	M21	M22
Typage HLA	X																	
Visite et NFS-plaquettes-AST/ALT-T Quick	X	X	X	X	X	X	X	X	X	X	X	X	X	X	X	X	X	X
Injection de vaccin		X		X		X						X						
ADN-VHB	X	X		X		X		X		X		X		X	X	X	X	
Sérologie VHB	X	X		X		X		X		X		X		X	X	X	X	
Immunologie	X	X	X		X		X						X				X	

Durée de participation de chaque patient : 24 mois (2 mois de pré-inclusion + 22 mois de traitement/suivi).

Références :

1. Michel, M.-L., H. L. Davis, M. Schleef, M. Mancini, P. Tiollais, and R. G. Whalen. 1995. DNA-mediated immunization to the hepatitis B surface antigen in mice: Aspects of the humoral response mimic hepatitis B viral infection in humans. *Proc. Natl. Acad. Sci, U.S.A. 92:5307.*

2. Tacket, C. O., M. J. Roy, G. Widera, W. F. Swain, S. Broome, and R. Edelman. 1999. Phase I safety and immune response studies of a DNA vaccine encoding hepatitis B surface antigen deliverd by a gene delivery device. *Vaccine 17:2826.*

3. Galibert, F., E. Mandart, F. Fitoussi, P. Tiollais, and P. Charnay. 1979. Nucleotide sequence of the hepatitis B virus genome (subtype ayw) cloned in E. coli. *Nature 281:646.*

4. Dubois, M.-F., C. Pourcel, S. Rousset, C. Chany, and P. Tiollais. 1980. Excretion of hepatitis B surface antigen particles from mouse cells transformed with cloned viral DNA. *Proc. Natl. Acad. Sci. USA 77:4549.*

5. Parker, S. E., F. Borellini, M. L. Wenk, P. Hobart, S. L. Hoffman, R. Hedstrom, T. Le, and J. A. Norman. 1999. Plasmid DNA malaria vaccine: tissue distribution and safety studies in mice and rabbits. *Hum Gene Ther 10:741.*

6. Davis, H. L., M.-L. Michel, and R. G. Whalen. 1993. DNA based immunization for hepatitis B induces continuous secretion of antigen and high levels of circulating antibody. *Hum. Molec. Genet 2:1847.*

7. Wang, R., D. L. Doolan, T. P. Le, R. C. Hedstrom, K. M. Coonan, Y. Charoenvit, T. R. Jones, P. Hobart, M. Margalith, J. Ng, W. R. Weiss, M. Sedegah, C. de Taisne, J. A. Norman, and S. L. Hoffman. 1998. Induction of antigen-specific cytotoxic T lymphocytes in humans by a malaria DNA vaccine. *Science 282:476.*

8. MacGregor, R. R., J. D. Boyer, K. E. Ugen, K. E. Lacy, S. J. Gluckman, M. L. Bagarazzi, M. A. Chattergoon, Y. Baine, T. J. Higgins, R. B. Ciccarelli, L. R. Coney, R. S. Ginsberg, and D. B. Weiner. 1998. First human trial of a DNA-based vaccine for treatment of human immunodeficiency virus type 1 infection: Safety and host response. *J. of Infect. Dis. 178:92.*

9. Calarota, S., G. Bratt, S. Nordlund, J. Hinkula, A. C. Leandersson, E. Sandstrom, and B. Wahren. 1998. Cellular cytotoxic response induced by DNA vaccination in HIV-1-infected patients. *Lancet 351:1320.*

10. Martin, T., S. E. Parker, R. Hedstrom, T. Le, S. L. Hoffman, J. Norman, P. Hobart, and D. Lew. 1999. Plasmid DNA malaria vaccine: the potential for genomic integration after intramuscular injection. *Hum Gene Ther 10:759.*

11. Davis, H. L., M. J. McCluskie, J. L. Gerin, and R. H. Purcell. 1996. DNA vaccine for hepatitis B: evidence for immunogenicity in chimpanzees and comparison with other vaccines. *Proc. Natl. Acad. Sci. USA 93:7213.*

12. Prince, A. M., R. Whalen, and B. Brotman. 1997. Successful nucleic acid based immunization of newborn chimpanzees against hepatitis B virus. *Vaccine 15:916.*

13. Wolff, J. A., R. W. Malone, P. Williams, W. Chong, G. Ascadi, A. Jani, and P. L. Felgner. 1990. Direct gene transfer into mouse muscle in vivo. *Science 247:1465.*

14. Tang, D., M. De Vit, and S. A. Johnston. 1992. Genetic immunization is a simple method for eliciting an immune response. *Nature 356:152.*

15. Michel, M.-L. 1997. Les vaccins à base d'ADN. *Virologie 1:283.*

16. Donnelly, J. J., J. B. Ulmer, J. W. Shiver, and M. A. Liu. 1997. DNA vaccines. *Annu. Rev. Immunol. 15:617.*

17. Mancini, M., H. L. Davis, P. Tiollais, and M.-L. Michel. 1996. DNA-based immunization against the envelope proteins of the hepatitis B virus. *J. Biotechnol. 44:47.*

18. Davis, H. L., M.-L. Michel, M. Mancini, M. Schleef, and R. G. Whalen. 1994. Direct gene transfer in skeletal muscle: plasmid DNA-based immunization against the hepatitis B virus surface antigen. *Vaccine 12:1503.*

19. Davis, H. L., M. Mancini, M.-L. Michel, and R. G. Whalen. 1996. DNA-mediated Immunization to Hepatitis B Surface Antigen: Longevity of Primary Response and Effect of Boost. *Vaccine 14:910.*

20. Davis, H. L., M. L. Michel, M. Mancini, M. Schleef, and R. G. Whalen. 1995. DNA-based immunization overcomes H-2 haplotype-restricted non-responsiveness to hepatitis B surface antigen in mice. In *Vaccines 95, vol. Molecular approches to the control of infectious diseases.* H. S. Ginsberg, F. Brown, and R. M. Chanocck, eds. Cold Spring Harbor Laboratory Press, New-York, p. 111.

21. Le Borgne, S., M. Mancini, R. Le Grand, M. Schleef, D. Dormont, P. Tiollais, Y. Rivière, and M.-L. Michel. 1998. In vivo induction of specific cytotoxic T lymphocytes in mice and rhesus macaques immunized with DNA vector encoding an HIV epitope fused with hepatitis B surface antigen. *Virology 240:304.*

22. Chow, Y. H., B. L. Chiang, Y. L. Lee, W. K. Chi, W. C. Lin, Y. T. Chen, and M. H. Tao. 1998. Development of Th1 and Th2 populations and the nature of immune

33. Bréchot, C. in: Rizzetto, M., Purcell, R.H., Gerin, J.L., Verme, G. (Editors). Molecular mechanisms of hepatitis B and C viruses related liver carcinogenesis. Viral hepatitis and liver disease. *Proceeding of the IX international meeting on hepatitis virus. Edizioni Minerva Medica, 1997, 490-508.*

34. Bréchot, C., Jaffredo, F., Lagorce, D., Gerken, G., Meyer zum Buschenfelde, K.H., Papakonstontinou, A., Hadziyannis, S., Rome, R., Colombo, M., Rodès, J., Bruix, J., Williams, R., Naoumov, N. Impact of HBV, HCV and GBV-C/HGV on hepatocellular carcinomas in Europe: results of an European concerted action. *J. Hepatol, 1998; 29:173-183.*

35. Chang MH, Chen CJ, Lai MS, Hsu HM, Wu TC, Kong MS et al. Universal hepatitis B vaccination in Taiwan and the incidence of hepatocellular carcinoma in children. *N Eng J Med* 1997; 336: 1855-9

36. Niederau K, Heintges T, Lange S, Goldmann G, Niederau CM, Mohr L, Häussinger G. Long-term follow-up of HBeAg-positive patients treated with interferon alfa for chronic hepatitis B. N Engl J Med 1996; 334: 1422-7.

37. Fazle Akbar S.M., Kajino K, Tanimoto K, Kurose K., Masumoto T., Michitaka K., Horiike N., Onji M. Placebo-controlled trial of vaccination with hepatitis B virus surface antigen in hepatitis B virus transgenic mice. *J Hepatol. 1997; 26: 131-137.*

38. Mancini M., Hadchouel M., Tiollais P., Pourcel C., Michel M-L. Induction of anti-hepatitis B surface antigen (HBsAg) antibodies in HBsAg producing transgenic mice: a possible way of cicumventing "nonresponse to HBsAg. *Journal of Medical Virology, 1993; 39: 67-74.*

39. Couillin I., Pol S., Mancini M., Driss F., Bréchot C., Tiollais P., Michel M-L. Specific vaccine therapy in chronic hepatitis B: induction of T cell proliferative responses specific for envelope antigens. *Journal of Infect Dis, 1999; 180: 15.*

40. Herroelen L, De Keyser J, Ebinger G. Central-nervous-system demyelination after immunisation with recombinant hepatitis B vaccine. *Lancet, 1991; 338: 1174-75.*

41. Zipp, F., Weil, J.G, Einhaupl, K.M. No increase in demyelinating diseases after hepatitis B vaccination. *Nature Medicine, 1999; 5: 964-5.*

42. Pol, S., Nalpas, B., Driss, F., Michel, M.L., Tiollais, P., Denis, J., Bréchot, C and a multicenter study group. Efficacy and limitations of a specific immunotherapy in chronic hepatitis B *(submitted).*

43. Liaw YF, Tai DI, Chu CM, Chen TJ. The development of cirrhosis in patients with chronic type B hepatitis: a prospective study. *Hepatology 1988; 8: 493-96.*

44. Fattovitch G, Brollo L, Giustina G, Noventa F, Pontisso P, Alberti A et al. Natural history and prognostic factors for chronic hepatitis B. *Gut 1991, 32: 294-98.*

45. Beasley RP, Lin CC, Hwang LY, Chien CS. Hepatocellular carcinoma and hepatitis B virus: a prospective study of 22707 men in Taiwan. *Lancet 1981; 1129-1133.*

46. Sheen IS, Liaw YF, Tai DI, CHU CM. Hepatic decompensation associated with hepatitis B e antigen clearance in chronic type B hepatitis. *Gastroenterology 1985; 89: 732-5.*

47. Liaw YF, Chu CM, Su IJ, Huang MJ, Lin DY, Chang-Chien CS. Clinical and histological events preceding hepatitis B e antigen seroconversion in chronic type B hepatitis. *Gastroenterology 1983; 84: 216-9.*

48 Pol S, Driss F, Michel M-L, Nalpas B, Berthelot P, Bréchot C. Specific vaccine therapy in chronic hepatitis B infection. *Lancet, 1994; 342 (letter).*

49. Wen Y-M, Wu X-H, Hu D-C, Zhang Q-P, Gno S-Q. Hepatitis B vaccine and anti-HBs complex as approach for vaccine therapy. *Lancet, 1995; 345: 1575-1576 (letter).*

50. Zoulim, F., Trépo, C. Drug therapy for chronic hepatitis B: antiviral efficacy and influence of hepatitis B virus polymerase mutations on the outcome of therapy. *J.Hepatol, 1998; 29: 151-168.*

ANNEXE 0

Notice d'information et formulaire de consentement

VACCINOTHERAPIE SPECIFIQUE PAR ADN NU
AU COURS DES HEPATITES CHRONIQUES B
Projet INSERM # RBM 99.026
Notice d'information – But de la recherche

Le Docteur ____________________ m'a proposé de participer à la recherche biomédicale intitulée : "Vaccinothérapie spécifique par ADN nu comme traitement des hépatites chroniques dues au virus de l'hépatite B".
L'INSERM, promoteur de cet essai, a contracté une assurance conformément à la loi.
Le médecin m'a précisé que j'étais libre d'accepter ou de refuser de participer à cette recherche.
Afin d'éclairer ma décision j'ai bien reçu et compris les informations suivantes :
Vous êtes suivi (e) dans notre service pour une hépatite chronique active due à une infection par le virus de l'hépatite B. Vous avez bénéficié successivement de traitements par l'interféron-α puis la lamivudine ; ces deux traitements sont en effet les traitements de référence pour l'hépatite B. Malgré cette thérapie, la multiplication virale persiste comme en témoigne la détection de l'ADN du virus dans le sérum.
Nous avons montré au cours des années précédentes que, à côté des traitements antiviraux classiques, la vaccination par le vaccin contre l'hépatite B de sujets porteurs chroniques du virus pouvait stimuler leurs défenses immunitaires contre le virus, réduire la multiplication virale et donc avoir un effet bénéfique. Ces études ont également montré les limites de cette approche, son efficacité, en utilisant les vaccins actuels, n'étant pas pleinement satisfaisante.
Parallèlement, est apparu le concept de la "vaccination par ADN" qui consiste en injections intra-musculaires d'une partie du génome du virus codant pour la protéine d'enveloppe virale (utilisée comme préparation vaccinale dans les vaccins actuels). Il a été démontré que l'injection intramusculaire de cet ADN permettait d'obtenir la synthèse de la protéine et la stimulation d'une réponse immunitaire contre cette protéine virale, aboutissant potentiellement à une vaccination. Certains résultats, obtenus chez l'animal, suggèrent que ce type de vaccination pourrait être plus efficace qu'une vaccination basée sur une stratégie conventionnelle et ce en particulier, chez des sujets chroniquement infectés par le virus.

Toute injection intra-musculaire peut donner des effets secondaires minimes (douleur, induration au point d'injection), mais il n'y a aucun risque de complication locale importante. Une augmentation des enzymes hépatiques est parfois observée au moment de l'arrêt de la multiplication virale, spontanée ou secondaire au traitement, plus ou moins associée à une insuffisance hépatique chez les patients initialement cirrhotiques ; toutefois dans les protocoles de vaccination déjà réalisés, elle n'entrainait pas d'aggravation significative à long terme. Par ailleurs, la vaccination par ADN est une nouvelle forme de traitement et vous bénéficierez donc d'un suivi prolongé afin de vérifier l'absence de complication génétique due à l'injection de l'ADN viral dans les cellules musculaires. Aucune des études réalisées jusqu'à présent chez l'animal ou chez l'homme n'a cependant montré la survenue de telles complications. Il n'y a également aucune donnée faisant suggérer un risque pour votre descendance.

Dans ces conditions nous vous proposons de participer à un premier protocole, visant à évaluer la tolérance et éventuellement l'efficacité de quatre injections de vaccin ADN (trois injections à deux mois d'intervalle puis une injection six mois après (soit au dixième mois du protocole) pour une étude dont la durée sera de vingt quatre mois. L'injection par voie intramusculaire sera effectuée comme pour une vaccination "standard". En fonction des résultats des prélèvements effectués à la visite de pré-inclusion (M-2), la participation à l'étude sera décidée et la première injection du vaccin aura lieu deux mois plus tard (M0). Des prélèvements sanguins seront ensuite effectués tous les mois permettant le suivi du bilan hépatique et de la multiplication virale (5-10

ml et à certaines dates 50 ml*) pendant les douze premiers mois, puis tous les trois mois après la fin de l'étude, la durée totale du protocole étant de 24 mois. Il vous est de plus demandé une contraception efficace, pour l'homme et la femme, pendant la première année de l'étude (soit jusqu'à deux mois après la dernière injection de vaccin).
La participation à cette étude est bénévole, mais le traitement peut avoir pour vous un bénéfice direct. Les résultats pourront vous être communiqués par votre médecin traitant de façon toutefois un peu différée.

* 7 Prélèvements de 50 ml à M-2, M0, M1, M3, M5, M11 et M21.

VACCINOTHERAPIE SPECIFIQUE PAR ADN NU
AU COURS DES HEPATITES CHRONIQUES B
Projet INSERM # RBM 99.026

Formulaire de consentement éclairé

Je soussigné (e) :_______________________________ (nom, prénom), certifie avoir lu et compris le document d'information qui m'a été remis.

J'ai eu la possibilité de poser toutes les questions que je souhaitais au Dr __________.
Je comprends les contraintes et les avantages liés à ma participation.
Je connais la possibilité qui m'est réservée d'interrompre le traitement à tout moment sans avoir à justifier ma décision mais je m'engage à informer le Dr__________. Cela ne remettra pas en cause la qualité des soins ultérieurs.
J'ai eu l'assurance que les décisions qui s'imposent pour ma santé seront prises à tout moment, conformément à l'état des connaissances et en fonction de l'évolution de ma propre maladie.
J'accepte que les données enregistrées à l'occasion de ce protocole puissent faire l'objet d'un traitement informatisé. J'ai bien noté que le droit d'accès prévu par la loi Informatique et Libertés (article 40), s'exerce à tout moment auprès du Dr__________ et que je pourrai exercer mon droit de rectification auprès de ce même médecin.
J'accepte que tout médecin ou scientifique impliqué dans le déroulement de cet essai, ainsi que le représentant des Autorités de Santé aient accès aux données qui me concernent dans le respect le plus strict de la confidentialité.
Mon consentement ne décharge en rien les organisateurs de l'étude de leurs responsabilités, je conserve tous mes droits garantis par la loi. Le retrait de l'étude ne me fait encourir aucune responsabilité.
J'accepte librement de participer à cette recherche dans les conditions précisées dans le document d'information.

Fait à____________________, le______________, Signature__________________.

Je soussigné (e) Dr________________ certifie avoir communiqué toute information utile concernant cette étude. Je m'engage à faire respecter les termes de cette note de consentement, conciliant le respect des droits et des libertés individuelles et les exigences d'un travail scientifique.

Fait à____________________, le______________, Signature__________________.

Ce projet a été soumis au **CCPPRB-Paris Necker** qui a rendu un **avis favorable le 7 novembre 2000.**
